# 67 national-level factors potentially related to the incidence of kidney replacement therapy across Europe

**DOI:** 10.1093/ndt/gfaf163

**Published:** 2025-08-28

**Authors:** Eva Pella, Rianne Boenink, Anneke Kramer, Kitty J Jager, Alberto Ortiz, Vianda S Stel

**Affiliations:** Nephrocare Hemodialysis Unit, Thessaloniki, Greece; ERA Registry, Department of Medical Informatics, Amsterdam UMC – location University of Amsterdam, Amsterdam, The Netherlands; Amsterdam Public Health, Quality of Care and Ageing & Later Life, Amsterdam, The Netherlands; ERA Registry, Department of Medical Informatics, Amsterdam UMC – location University of Amsterdam, Amsterdam, The Netherlands; Amsterdam Public Health, Quality of Care and Ageing & Later Life, Amsterdam, The Netherlands; ERA Registry, Department of Medical Informatics, Amsterdam UMC – location University of Amsterdam, Amsterdam, The Netherlands; Amsterdam Public Health, Quality of Care and Ageing & Later Life, Amsterdam, The Netherlands; Department of Nephrology and Hypertension, IIS-Fundacion Jimenez Diaz UAM, Madrid, Spain; RICORS2040, Madrid, Spain; ERA Registry, Department of Medical Informatics, Amsterdam UMC – location University of Amsterdam, Amsterdam, The Netherlands; Amsterdam Public Health, Quality of Care and Ageing & Later Life, Amsterdam, The Netherlands

**Keywords:** aging, disease burden, heterogeneity, kidney replacement therapy, physical activity

## Abstract

**Background:**

Kidney replacement therapy (KRT) incidence varies considerably across European countries. We aimed to provide an overview of factors potentially related to KRT incidence for all individual European countries and across low, middle and high KRT incidence countries and to describe the relationship between these factors and KRT incidence.

**Methods:**

We obtained unadjusted KRT incidence rates from the European Renal Association (ERA) Registry annual reports and studies. Countries were divided into low [0–100 per million population (pmp)], middle (100–200 pmp) and high (>200 pmp) KRT incidence countries. Online sources were searched for information on factors potentially related to KRT incidence including geographic, socioeconomic, sociocultural and health-related factors, and factors related to chronic kidney disease (CKD) and national capacity for CKD prevention. Univariate linear or polynomial regression were used to examine whether factors and KRT incidence were related, with the R coefficient as metric of correlation strength. Significant factors were also evaluated separately in less affluent and wealthy countries.

**Results:**

Thirty-eight European countries were included, and divided into 12 low, 21 middle and 5 high KRT incidence countries. Among 67 factors evaluated, the number of practicing physicians (R = 0.374, *P* = .023), the population density (R = 0.508, *P* = .001), the median age at KRT initiation (R = 0.549, *P* = .001), the percentages of CKD-attributed deaths (R = 0.418, *P* = .038) and disability-adjusted life years (R = 0.420, *P* = .010), and the physical inactivity prevalence (R = 0.569, *P* < .001) were significantly positively correlated with KRT incidence. These findings were consistent among less affluent countries, while median age at KRT initiation was the only significant factor among wealthy countries (R = 0.889, *P* < .001). After multiple testing correction, median age at KRT initiation, physical inactivity prevalence and population density remained correlated with KRT incidence.

**Conclusions:**

These findings may be a first step for policy makers, stakeholders and nephrologists to optimize healthcare (planning) regarding KRT initiation and reduce KRT incidence disparities.

## Graphical Abstract

**Graphical Abstract:**
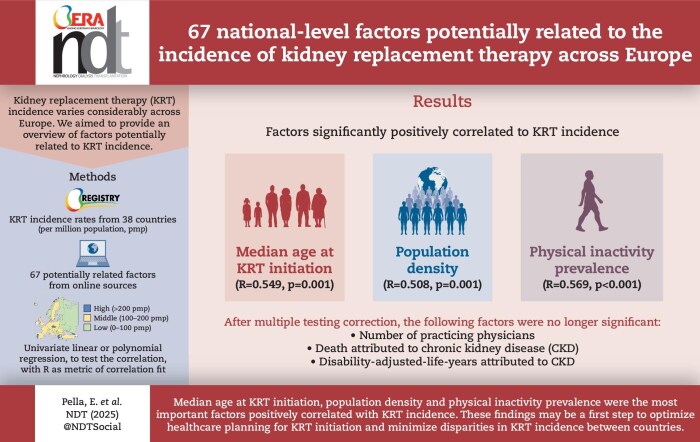

KEY LEARNING POINTS **What was known:**

- Kidney replacement therapy (KRT) incidence varies globally, but also across European countries.
- A higher KRT incidence could be explained by a higher prevalence of (risk factors for) chronic kidney disease (CKD) in the general population, a higher progression from CKD to kidney failure and/or a higher access to KRT; macroeconomic factors could influence all the aforementioned categories.

**This study adds:**

- The current study presents the most extensive and up-to-date overview of factors potentially related to the incidence of KRT across European countries; it includes 67 geographical, socioeconomic, sociocultural, health- and CKD-related factors, and factors related to the national capacity for CKD prevention for 38 European countries individually.
- We found that the median age at KRT initiation, physical inactivity prevalence and population density were the most important factors significantly positively correlated with KRT incidence.

**Potential impact:**

- Our findings could serve as a source for individual countries to compare their KRT incidence and explore the factors potentially underlying their KRT incidence with that of other countries.
- The results of this study could be used by policy makers, stakeholders and nephrologists to optimize healthcare (planning) regarding KRT initiation and in turn reduce disparities in KRT incidence.

## INTRODUCTION

Large differences exist in the kidney replacement therapy (KRT) incidence globally [1, 2], but also across European countries [3]. In 2021, KRT incidence rates ranged from 53 per million population (pmp) in Ukraine to 283 pmp in Cyprus, with one-third of European countries reporting values <100 pmp and one-tenth >200 pmp [3].

Several factors have been reported to contribute to these differences in KRT incidence. They could be categorized in factors related to the prevalence of chronic kidney disease (CKD) and its major risk factors [4–6], the progression of CKD and the speed of this progression (e.g. early intervention and treatment) [5, 7, 8], and the access to KRT (e.g. referral policies and timing of KRT initiation) [5, 9, 10]. Interestingly, macroeconomic factors affect all the aforementioned categories and may contribute crucially to international differences in KRT incidence as they could influence sociocultural, infrastructural and educational development [5, 10, 11].

Previous studies have attempted to explain the differences in KRT incidence in Europe. Notwithstanding the value of these studies, they evaluated a limited range of factors, such as comorbidities [12, 13], clinical practice [12, 14–17] or macroeconomic factors [12, 16]. Moreover, most studies included only a few countries [15, 18, 19] or different regions within a country [14, 16, 17, 20, 21]. In addition, several recently published studies have reported differences in various factors that may have contributed to international differences in KRT incidence in Europe, such as capacity of KRT management and prevalence of CKD risk factors, but they did not evaluate if these factors were related to KRT incidence [4, 11, 22–24]. As such, a comprehensive and up-to-date overview of factors potentially related to KRT incidence and their correlations with KRT incidence across all European countries is still lacking.

Therefore, the aim of this study was to provide an overview of a wide range of factors potentially related to KRT incidence for all individual European countries and across low, middle and high KRT incidence countries. In addition, we aimed to describe the relationship between these factors and the incidence of KRT.

## MATERIALS AND METHODS

### Study design and data collection

This is a cross-sectional, ecological study based on online available national-level data. Supplementary data, Table S1 provides an overview of the sources used to extract KRT incidence rates and data on factors potentially related to KRT incidence.

The European Renal Association (ERA) Registry annually collects data on patients receiving KRT from national and regional renal registries in Europe [3]. Unadjusted KRT incidence rates were obtained from the 2021 ERA Registry annual report [3] for most countries and from earlier ERA Registry annual reports [25–27] for Hungary, Russia, Bulgaria and Georgia. The ERA Registry data covers the complete general population for most countries, except for Croatia (85%), Italy (35%), Slovakia (82%) and Ukraine (64%). For Germany, the ERA Registry does not receive data, and therefore an estimated KRT incidence rate was obtained from a former ERA Registry study [5]. KRT incidence pmp was defined as the number of patients initiating KRT due to kidney failure during a year, divided by the general population in that year and multiplied by 1 million [3].

Factors potentially related to KRT incidence were grouped into three main categories: (i) geographical, socioeconomic, sociocultural and health-related factors, (ii) factors related to CKD, and (iii) factors related to national capacity for CKD prevention. These factors were obtained for each participating country using online databases from renowned institutions [28–38] such as the World Health Organization and previously published articles [1, 5, 39, 40] (Supplementary data, Table S1). When available, 2021 data were extracted, otherwise, the latest data available were used. The online databases were assessed from May to July of 2024. Almost 100 unadjusted factors were initially identified. After discussing with experts, factors with incomplete data or overlapping meaning were removed and 67 factors were selected.

### Statistical analysis

The distribution of continuous variables was expressed as mean (standard deviation) or median (interquartile range), whereas for categorical factors this was expressed as counts per total (*n*/*N*) and percentages. For continuous variables, we used visual interpretation to determine the pattern of the univariate relationship between each factor and KRT incidence, and linear or polynomial regression to model linear or curvilinear univariate relationships using data from all individual countries [41, 42]. We did not perform multivariable analysis, as our aim is hypothesis-generating and not intended to establish causality. The R coefficient was used as metric of correlation strength, representing the Pearson's (for normally distributed data) or Spearman's (for non-normally distributed data) correlation coefficient for simple linear relationships and the multiple correlation coefficient from the polynomial regression for curvilinear relationships [43]. Countries were divided into low (0–100 pmp), middle (100–200 pmp) and high (>200 pmp) KRT incidence countries. These cut-off values were chosen to enable focusing on the few countries with relatively high incidence rates. As an exploratory secondary analysis, the significant findings were evaluated separately among wealthy [gross domestic product (GDP) per capita >$40 000] and less affluent (GDP per capita <$40 000) countries. We selected this cut-off value in alignment with the reported average values of GDP per capita in 2021 (i.e. $38 721 in the European Union [28] and $42 527 in Euro area [28]). Statistical analysis was performed with SPSS 28 (SPSS Inc, Chicago, IL, USA). *P*-values <.05 were considered statistically significant. We also checked which correlations remained statistically significant after Bonferroni correction for multiple testing using a stricter *P*-value threshold set at .0013 [44].

## RESULTS

A total of 38 European countries were included in this study, and divided into 12 low (0–100 pmp), 21 middle (100–200 pmp) and 5 high (>200 pmp) KRT incidence countries (Supplementary data, Fig. S1). Supplementary data, Table S2 includes all 67 factors potentially related to KRT incidence and their values by country.

### Geographical, socioeconomic, sociocultural and health-related factors

**Figure 1:**
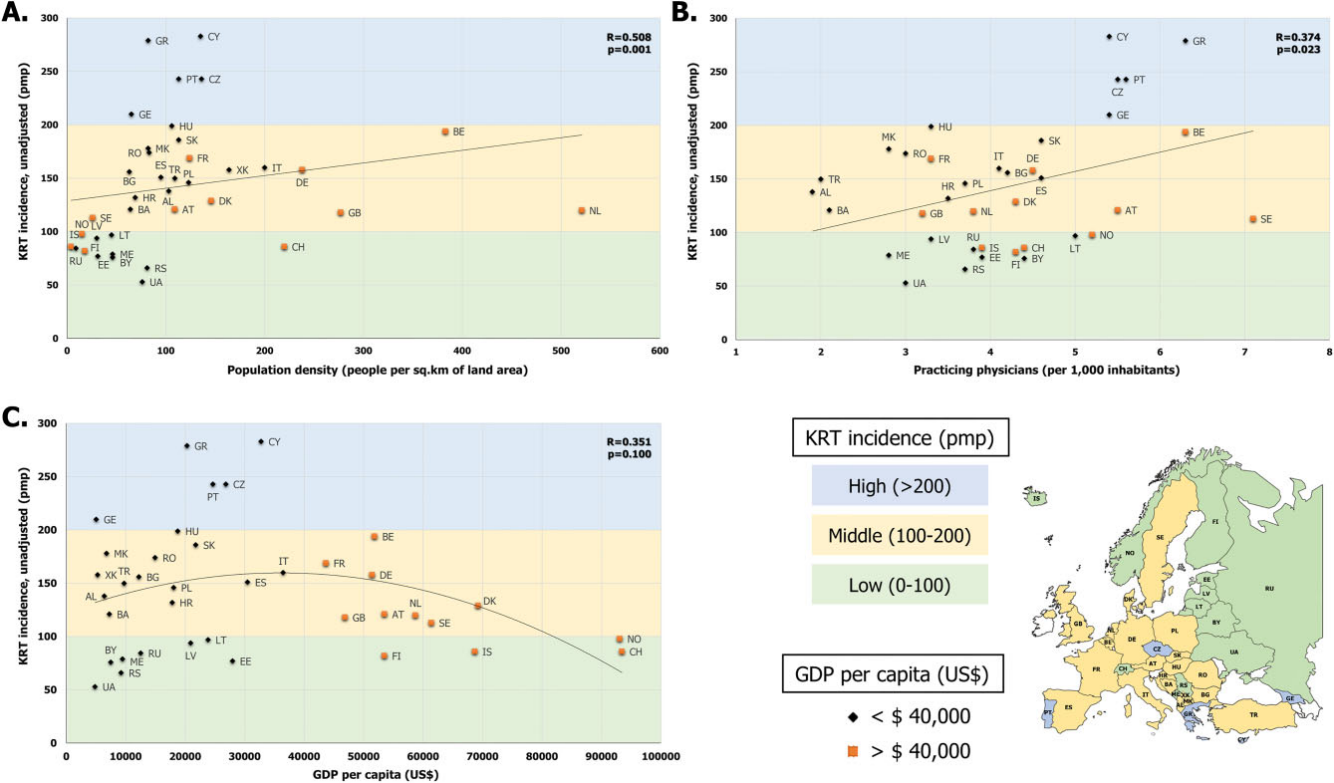
Scatterplots presenting the relationship between KRT incidence (pmp) and: (**A**) population density (people per km^2^ of land area), (**B**) practicing physicians (per 1000 inhabitants) and (**C**) GDP per capita (US$). Countries are stratified by KRT incidence, in low (0–100 pmp, green color), middle (100–200 pmp, yellow color) and high- (>200 pmp, blue color) incidence groups, and by GDP per capita, in wealthy (GDP >$40 000, yellow square marker) and less affluent (GDP <$40 000, black rhombus marker) country groups.

Table 1 presents the geographical, socioeconomic, sociocultural and health-related factors per KRT incidence group of countries, and the relationship (R coefficient) of each factor with KRT incidence among all included countries. Scatterplots for these factors and KRT incidence are shown in Fig. 1 and Supplementary data, Figs S2–S5.

**Table 1:** Geographical, socioeconomic, sociocultural and health-related factors and their relationship with KRT incidence among all countries, and among low, middle and high KRT incidence countries.

	KRT incidence (pmp) group				Relationship (all individual countries)	
Factor	Low (0–100 pmp) (12 countries)	Middle (100–200 pmp) (21 countries)	High (>200 pmp) (5 countries)	All (38 countries)	R coefficient	*P*-value
KRT incidence (pmp)	82 (13)	151 (26)	252 (30)	142 (58)		
Geographical and socioeconomic factors						
Population density (people per km^2^ of land area)	38 (16–69)	109 (83–182)	113 (74–136)	89 (46–135)	0.508	**.001**
General population aged 65+ years (% of total population)	18.5 (16.3–21.0)	19.0 (17.5–20.5)	20.0 (14.5–23.0)	19.0 (16.8–21.0)	0.211^a^	.450
Rural population (% of total population)	25.7 (10.0)	27.9 (14.4)	28.0 (6.3)	27.1 (12.2)	0.071^a^	.919
GDP per capita (US$)	22 390 (9291–64 909)	21 768 (10 981–51 639)	24 661 (12 667–29 785)	22 809 (9674–51 533)	0.351^a^	.100
Sociocultural factors						
Education Index	0.883 (0.066)	0.848 (0.077)	0.864 (0.054)	0.861 (0.071)	0.146^a^	.692
Innovation Index	41.6 (12.0)	43.8 (11.4)	38.7 (7.4)	42.4 (11.0)	0.334^a^	.134
Human Development Index	0.873 (0.072)	0.876 (0.062)	0.871 (0.037)	0.874 (0.061)	0.390^a^	.060
Health indicators						
Life expectancy (years)						
At 65–69 years	16.0 (14.1–20.9)	18.0 (15.6–20.3)	19.0 (15.5–19.6)	17.0 (15.2–20.3)	0.257^a^	.312
At 40–44 years	35.4 (33.7–43.9)	39.8 (36.2–42.8)	41.0 (36.1–42.2)	38.2 (35.0–42.7)	0.316^a^	.168
Mortality rate						
All causes (death rate per 100 000 population)	1238 (443)	1217 (356)	1215 (300)	1224 (370)	0.189^a^	.549
Cardiovascular (death rate per 100 000 population)	544 (288)	426 (248)	400 (148)	462 (253)	0.211^a^	.471
Healthcare factors						
Practicing physicians (per 1000 inhabitants)	4.0 (0.7)	3.9 (1.4)	5.6 (0.4)	4.2 (1.2)	0.374	**.023**
Practicing nurses (per 1000 inhabitants)	10.2 (6.3–18.1)	7.6 (5.9–11.2)	5.9 (4.2–8.4)	7.6 (5.9–11.3)	0.356^a^	.100
Nephrologists (pmp)	26.3 (11.8)	26.9 (12.2)	30.9 (5.6)	27.3 (11.3)	0.328^a^	.144
Nephrology trainees (pmp)	4.4 (3.8)	5.2 (2.8)	6.7 (5.3)	5.2 (3.5)	0.201^a^	.495
Salary of nephrologists per year (US$)	125 347 (117 658)	117 260 (76 789)	91 988 (9072)	116 468 (86 377)	0.205^a^	.480
Current health expenditure (% of GDP)	8.8 (1.7)	9.3 (2.4)	9.1 (1.4)	9.1 (2.1)	0.045^a^	.966
Healthcare Access and Quality Index	85 (9)	86 (8)	84 (10)	85 (9)	0.143^a^	.704
Primary care physician referral for access secondary care (required)	4/12 (33%)	8/21 (38%)	1/5 (20%)	13/38 (34%)		
Total number of renal centers of country (pmp)	9.2 (7.1)	8.1 (3.9)	11.2 (5.2)	8.9 (5.3)	0.363^a^	.090
Travel time to healthcare facilities						
% of population beyond 30 min (optimal speed, motorized transport)	9 (7–22)	1 (0–12)	4 (0–13)	5 (0–16)	0.272^a^	.270

Values are expressed as: (i) mean (standard deviation) or median (interquartile range) according to their distribution (continuous factors), or (ii) counts and percentages (categorical factors).

The R coefficient represents the Pearson's (for normally distributed data) or Spearman's (for non-normally distributed data) correlation coefficient for simple linear relationships and the multiple correlation coefficient for curvilinear relationships including all individual countries.

^a^Curvilinear relationships.

Population density (R = 0.508, *P* = .001) was statistically significantly related to KRT incidence (Fig. 1A). High KRT incidence countries reported more population per square-meter than almost all low KRT incidence countries. This correlation remained significant after multiple testing correction. Interestingly, the countries with the highest population density were found among the wealthy middle KRT incidence countries. In addition, the number of practicing physicians (i.e. general practitioners and specialists) per 1000 inhabitants (R = 0.374, *P* = .023) was statistically significantly related to KRT incidence (Fig. 1B). High KRT incidence countries reported more practicing physicians per 1000 inhabitants (from 5.4 in Georgia and Cyprus to 6.3 in Greece) than all low (from 2.8 in Montenegro to 5.2 in Norway) and the majority of middle KRT incidence countries. This correlation did not remain statistically significant after multiple testing correction. Although the GDP per capita was not statistically significantly related to KRT incidence (inverted U-shaped curve, R = 0.351, *P* = .100), among low KRT incidence countries we observed a cluster of less affluent eastern European countries and a cluster of wealthy western European countries (Fig. 1C).

The remaining geographical, socioeconomic, sociocultural and health-related factors studied did not show a correlation with KRT incidence (Table 1 and Supplementary data, Figs S2–S5).

### CKD-related factors

**Figure 2:**
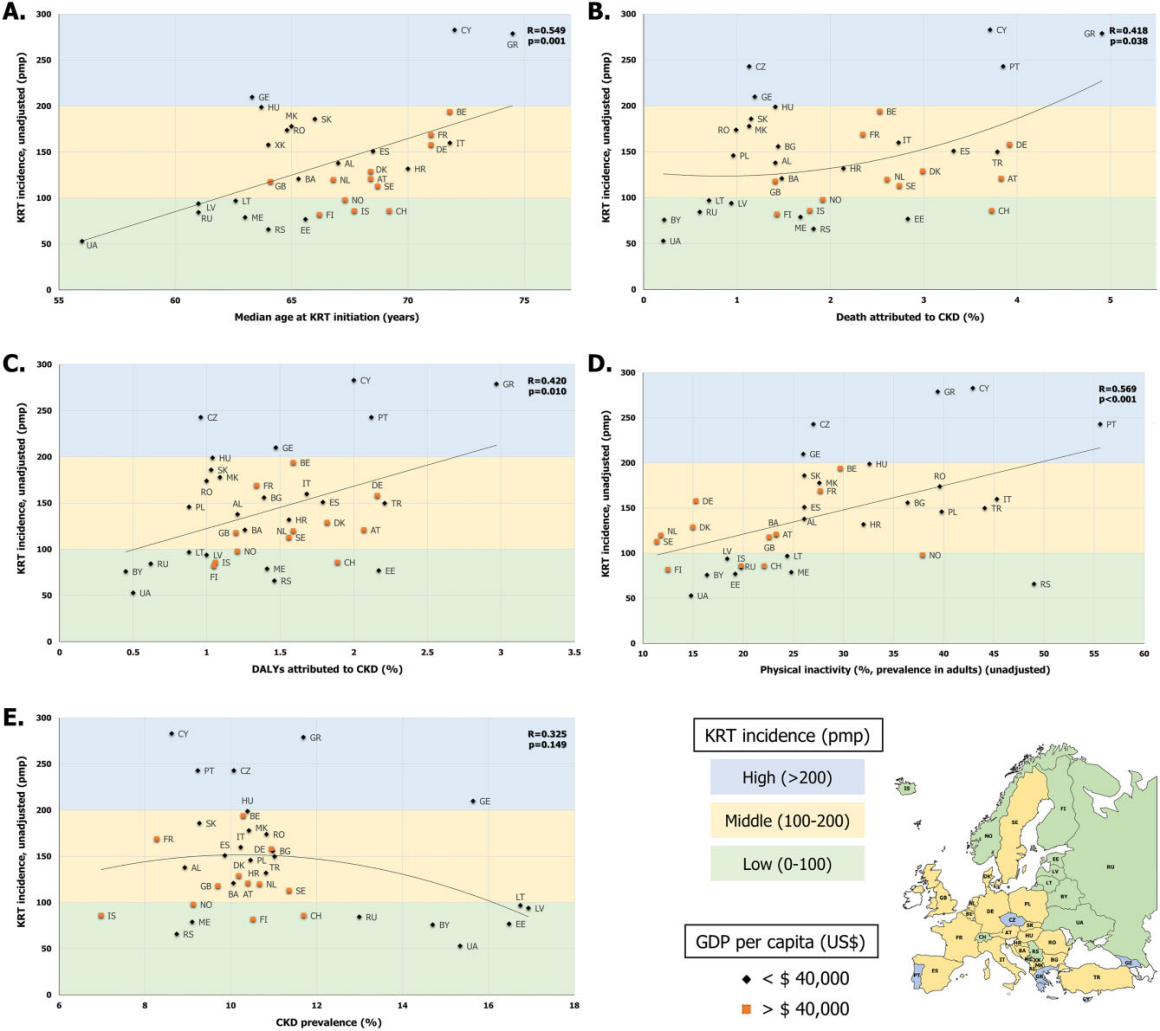
Scatterplots presenting the relationship between KRT incidence (pmp) and: (**A**) median age at KRT initiation (years), (**B**) death attributed to CKD (%), (**C**) DALYs attributed to CKD (%), (**D**) physical inactivity (%, prevalence in adults) and (**E**) CKD prevalence (%). Countries are stratified by KRT incidence, in low (0–100 pmp, green color), middle (100–200 pmp, yellow color) and high (>200 pmp, blue color) incidence groups, and by GDP per capita, in wealthy (GDP >$40 000, yellow square marker) and less affluent (GDP <$40 000, black rhombus marker) country groups.

The CKD-related factors and their relationship with KRT incidence are presented in Table 2, Fig. 2 and Supplementary data, Figs S6–S9.

**Table 2:** CKD-related factors and their relationship with KRT incidence among all countries, and among low, middle and high KRT incidence countries.

	KRT incidence (pmp) group				Relationship (all individual countries)	
Factor	Low (0–100 pmp) (12 countries)	Middle (100–200 pmp) (21 countries)	High (>200 pmp) (5 countries)	All (38 countries)	R coefficient	*P*-value
KRT incidence (pmp)	82 (13)	151 (26)	252 (30)	142 (58)		
CKD						
CKD prevalence (%)	12.4 (9.1–16.2)	10.4 (9.9–10.8)	10.1 (8.9–13.7)	10.4 (9.5–11.5)	0.325^a^	.149
Median age at KRT initiation (years)	64.0 (3.8)	67.6 (2.8)	69.9 (5.9)	66.6 (3.9)	0.549	**.001**
Death attributed to CKD (%)	1.6 (0.6–1.9)	2.3 (1.4–2.9)	3.7 (1.2–4.4)	1.8 (1.1–2.9)	0.418^a^	**.038**
DALYs attributed to CKD (%)	1.1 (0.5)	1.5 (0.4)	1.9 (0.8)	1.4 (0.5)	0.420	**.010**
Risk factors for CKD						
Diabetes (%, prevalence in adults)	8.5 (6.9–9.7)	8.5 (6.9–10.0)	9.7 (8.2–11.4)	8.6 (7.0–9.9)	0.209^a^	.470
Hypertension (%, prevalence in adults)	45.3 (8.7)	43.8 (8.1)	42.0 (6.5)	44.0 (8.0)	0.193^a^	.523
Obesity (%, prevalence in adults)	24.9 (5.0)	25.2 (7.9)	31.2 (5.5)	25.9 (7.0)	0.363^a^	.090
Smoking (%, prevalence in adults)	21.1 (7.5)	22.1 (6.7)	24.8 (3.9)	22.1 (6.6)	0.292^a^	.229
Physical inactivity (%, prevalence in adults)	19.8 (16.9–24.7)	26.9 (22.8–35.5)	39.4 (26.5–49.3)	26.1 (19.5–37.2)	0.569	**<.001**
Cardiovascular disease (%, prevalence in adults)	15.7 (2.1)	16.0 (2.3)	14.9 (4.1)	15.8 (2.5)	0.389^a^	.062
Salt intake (g/day)	8.1 (7.1–9.4)	9.2 (8.2–12.8)	8.8 (8.4–11.0)	8.5 (8.0–12.8)	0.253^a^	.326
Altitude (by country) (m)	318 (124–589)	414 (177–684)	430 (232–965)	422 (169–615)	0.102^a^	.832
Altitude (by most populated city of each country) (m)	78 (13–173)	97 (31–294)	220 (103–431)	110 (28–250)	0.195^a^	.507
CCM						
Capacity for CCM (generally available)	8/12 (67%)	16/21 (76%)	2/5 (40%)	26/38 (68%)		
EDITH study: CCM (among kidney failure patients)						
Patients who were offered CCM (%)	7.5 (2.8–10.0)	5.0 (3.5–9.0)	4.0 (3.0–5.0)	5.0 (3.0–10.0)	-0.375	.054
Macroeconomic factors related to CKD						
Annual cost of kidney replacement therapy components per patient						
HD (US$)	20 208 (15 564–55 874)	24 031 (16 400–61 468)	47 892 (108,69–77 572)	23 992 (16 290–63 962)	0.224^a^	.417
PD (US$)	21 198 (17 951–30 094)	27 206 (19 761–52 599)	29 737 (15 054–46 056)	26 851 (18 174–41 307)	0.240^a^	.366
Kidney transplant (first year) (US$)	64 475 (49 551)	55 500 (40 057)	42 520 (33 425)	55 331 (39 462)	0.301^a^	.489
Kidney transplant (later years) (US$)	18 773 (16 830)	20 565 (19 040)	15 469 (12 606)	19 068 (16 393)	0.211^a^	.761
HD/PD cost ratio	0.95 (0.79–1.36)	0.90 (0.74–1.41)	1.16 (0.66–2.19)	0.92 (0.77–1.43)	0.308^a^	.183
Free public funding for						
Non-dialysis CKD	5/12 (42%)	15/21 (71%)	3/5 (60%)	23/38 (61%)		
KRT modality						
HD	8/12 (67%)	16/21 (76%)	4/5 (80%)	28/38 (74%)		
PD	10/12 (83%)	18/21 (86%)	4/5 (80%)	32/38 (84%)		
Kidney transplantation	9/12 (75%)	17/21 (81%)	4/5 (80%)	30/38 (79%)		
Free public funding for medications						
CKD patients not on dialysis	3/12 (25%)	6/21 (29%)	1/5 (20%)	10/38 (26%)		
All dialysis patients	5/12 (42%)	9/21 (43%)	2/5 (40%)	16/38 (42%)		
All transplant patients	6/12 (50%)	8/21 (38%)	3/5 (60%)	45 (45%)		

Values are expressed as: (i) mean (standard deviation) or median (interquartile range) according to their distribution (continuous factors), or (ii) counts and percentages (categorical factors).

The R coefficient represents the Pearson's (for normally distributed data) or Spearman's (for non-normally distributed data) correlation coefficient for simple linear relationships and the multiple correlation coefficient for curvilinear relationships including all individual countries.

^a^Curvilinear relationships.

CCM, conservative care management; HD, hemodialysis; PD, peritoneal dialysis.

Overall, the median age at KRT initiation (R = 0.549, *P* = .001), the percentage of CKD attributed deaths (inverted U-shaped curve, R = 0.418, *P* = .038), the disability-adjusted life years (DALYs) attributed to CKD (R = 0.420, *P* = .010) and the physical inactivity prevalence (R = 0.569, *P* < .001) were statistically significantly related to KRT incidence. When using the Bonferroni-adjusted *P*-value threshold, only median age at KRT initiation and physical inactivity prevalence remained statistically significantly related to KRT incidence. The highest median age at KRT initiation was reported among the high KRT incidence countries (>72 years in Greece and Cyprus), while a median age <70 years was observed in all low KRT incidence countries (Fig. 2A). For the proportion of deaths and DALYs attributed to CKD, the highest values (4.9% and 3.0%, respectively) were observed in Greece (high KRT incidence country) and the lowest in Belarus and Ukraine (low KRT incidence countries) (∼0.2% and ∼0.5%, respectively; Fig. 2B and 2C). Finally, the CKD risk factor physical inactivity was highly prevalent (>39%) in the majority of high KRT incidence countries, with Portugal reporting the highest value (56%; Fig. 2D).

Although CKD prevalence among the general population was not statistically significantly related to KRT incidence (inverted U-shaped curve, R = 0.325, *P* = .149), among low KRT incidence countries, we observed both the highest (16.9% in Latvia) and the lowest (7% in Iceland) CKD prevalence (Fig. 2E). The remaining risk factors for CKD, factors regarding the capacity of offering conservative care management (CCM), and all macroeconomic factors studied were not correlated with KRT incidence (Table 2 and Supplementary data, Figs S6–S9).

### National capacity for CKD prevention

Table 3 presents the availability of assessments for early diagnosis of CKD and its risk factors at the primary healthcare level, pharmacological agents for attenuating CKD progression in the public health sector and policies, strategies and action plans for CKD prevention.

**Table 3:** The availability of factors related to national capacity for CKD prevention potentially related to KRT incidence among all countries, and among low, middle and high KRT incidence countries.

	KRT incidence (pmp) group			
Factor	Low (0–100 pmp) (12 countries)	Middle (100–200 pmp) (21 countries)	High (>200 pmp) (5 countries)	All (38 countries)
KRT incidence (pmp)	82 (13)	151 (26)	252 (30)	142 (58)
Access to early diagnosis of CKD and risk factors (availability of procedures) at the primary healthcare level				
Blood pressure measurement	12/12 (100)	19/21 (90)	5/5 (100)	36/38 (95)
Diabetes testing (by HbA1c)	12/12 (100)	17/21 (81)	4/5 (80)	33/38 (87)
Cardiovascular risk stratification in 50% or more primary healthcare facilities	11/12 (92)	16/21 (76)	3/5 (60)	30/38 (79)
Diabetes testing (by blood glucose measurement, oral glucose tolerance tests)	12/12 (100)	19/21 (90)	5/5 (100)	36/38 (95)
Urine testing for albumin	11/12 (92)	18/21 (86)	5/5 (100)	34/38 (89)
Availability of pharmacological agents in the public health sector				
Angiotensin-converting enzyme inhibitors	12/12 (100)	19/21 (90)	4/5 (80)	35/38 (92)
Angiotensin II receptor blockers	12/12 (100)	18/21 (86)	4/5 (80)	34/38 (89)
Metformin	12/12 (100)	19/21 (90)	4/5 (80)	35/38 (92)
Insulin	12/12 (100)	17/21 (81)	4/5 (80)	33/38 (87)
Beta-blockers	12/12 (100)	19/21 (90)	4/5 (80)	35/38 (92)
Calcium channel blockers	12/12 (100)	19/21 (90)	4/5 (80)	35/38 (92)
Policies, strategies and action plans				
National target on dietary salt	4/12 (33)	5/21 (24)	0/5 (0)	9/38 (24)
Any policies to reduce population salt consumption	12/12 (100)	18/21 (86)	5/5 (100)	35/38 (92)
Operational policy/strategy/action plan for diabetes	9/12 (75)	16/21 (76)	3/5 (60)	28/38 (74)
Implementation of physical activity public awareness program	12/12 (100)	20/21 (95)	5/5 (100)	37/38 (97)
Existence of evidence-based national guidelines/protocols/standards for the management of:				
Diabetes	12/12 (100)	20/21 (95)	5/5 (100)	37/38 (97)
Overweight/obesity	10/12 (83)	13/21 (62)	3/5 (60)	26/38 (68)
Major noncommunicable diseases through a primary care approach	11/12 (92)	17/21 (81)	4/5 (80)	32/38 (84)
Physical inactivity	9/12 (75)	14/21 (67)	2/5 (40)	25/38 (66)

Values are expressed as counts and percentages (categorical factors).

HbA1c, hemoglobin A1c.

Assessments for early diagnosis of CKD and its risk factors (e.g. urine testing for albumin) at the primary healthcare level and pharmacological agents (e.g. angiotensin-converting enzyme inhibitors) for attenuating CKD progression in the public health sector were generally available among almost all participating European countries. Guidelines for diabetes management were also reported in almost all European countries. However, in one-third of the countries we observed gaps in the national availability of guidelines for the management of other main risk factors for CKD such as overweight/obesity and physical inactivity, including Greece and Cyprus from the high KRT incidence countries (Supplementary data, Fig. S10). Finally, a national target on salt intake ranged from only 50% of wealthy low KRT incidence countries to none of the high KRT incidence countries (Supplementary data, Fig. S11).

### Secondary analysis: wealthy and less affluent countries

Supplementary data, Table S3 and Fig. S12 present the factors significantly related to KRT incidence separately in wealthy and less affluent countries. All factors remained significantly related to KRT incidence among less affluent countries, whereas only median age at KRT initiation remained significantly related to KRT incidence among wealthy countries.

## DISCUSSION

The current study presents the most extensive up-to-date overview of factors potentially related to the incidence of KRT across European countries. It includes 67 geographical, socioeconomic, sociocultural, health- and CKD-related factors, and factors related to the national capacity for CKD prevention for 38 European countries. We aimed to evaluate the univariate relationship between each of these factors and KRT incidence, with a focus on high KRT incidence countries, and not to prove causality. As such, the rationale in the selection of the factors was not necessarily explanatory and inevitably some factors may be both the cause and the consequence of differences in KRT incidence. In the following paragraphs we discuss the statistically significant relationships between the studied factors and KRT incidence. We also discuss observations on well-known factors that affect KRT incidence, even when no significant relationship was found in this study. Notably, all significant correlations were consistent among less affluent countries and only median age at KRT initiation among wealthy countries.

### Geographical, socioeconomic, sociocultural and health-related factors

We observed that high KRT incidence countries reported higher population density than almost all low KRT incidence countries. In high KRT incidence countries, factors like air pollution, sedentary lifestyle, limited green space and unhealthy dietary habits, common in densely populated areas, may contribute to higher rates of cardiovascular disease, physical inactivity and obesity—risk factors for CKD—and may explain KRT incidence differences [45]. High population density may also mean access to closer dialysis facilities that may facilitate the uptake of KRT, especially in the elderly. Wealthy countries may invest more in healthcare systems, infrastructure, sanitation and access to medical care which may mitigate the effect of high population density on CKD progression and incidence of KRT in the wealthy middle KRT incidence countries [46]. High KRT incidence countries reported more practicing physicians (i.e. general practitioners and specialists) per 1000 inhabitants than all low KRT incidence countries. Medical professionals in high KRT incidence countries may have the intention, as it may be part of their culture, to treat older patients to the fullest extent and for as long as needed, which could lead to an increasing demand for physicians. Additionally, the high prevalence of CKD and its risk factors in the general population among high KRT incidence countries could also create demand for medical doctors and nephrologists [31]. Moreover, in almost all high KRT incidence countries referral from a general practitioner was not mandatory for access to secondary care, including nephrologist consultation [38]. As such, our findings suggest that direct access to specialist care might be associated with a higher KRT incidence. Information on the number of primary care physicians and data on early or late referral to a nephrologist would add valuable insight to international differences in KRT incidence, but these data were not available for all included countries [47–50].

Although the relationship between GDP per capita and KRT incidence (inverted U-shaped curve) was not significant, among low KRT incidence countries we observed a cluster with less affluent eastern European countries and a cluster with wealthy western European countries suggesting that the national economic status may affect KRT incidence differences. For example, patients in mainly less affluent countries might have limited access to KRT, due to macroeconomic reasons, while in wealthy countries, there may be fewer people with kidney failure who are in need of KRT, as more resources might have been invested in CKD prevention [10].

### CKD-related factors

The median age at KRT initiation was one of the factors that remained significantly related to KRT incidence after multiple testing correction. The highest value (>72 years) in Europe was reported in two high KRT incidence countries (Greece and Cyprus). As mentioned above, in high KRT incidence countries referral from a general practitioner was not mandatory for access to secondary care, which potentially lead to a higher percentage of older patients examined by nephrologists, and referred to start KRT. Although we did not find significant relationships, we observed higher life expectancy and lower all-cause and cardiovascular mortality rates in the general population in high KRT incidence countries than in middle and low KRT incidence countries [29, 51, 52]. As such, among high KRT incidence countries a higher percentage of people may eventually need KRT as they may live longer and they may progress to kidney failure more often. In addition, Cordero *et al*. recently reported that some of the high KRT incidence countries (Greece and Cyprus) appear to have a more open attitude towards offering KRT in the elderly population than in other European countries [53, 54]. Finally, the limited availability of CCM in Greece and Cyprus could also explain our finding. In Greece for example, although CCM seemed to be acceptable as practice for kidney failure management [31], patients were usually not offered CCM [5].

Although the prevalence of CKD was not statistically significantly related to KRT incidence, the relationship highlights again the heterogeneity among low KRT incidence countries. Our observation that eastern European countries have a higher CKD prevalence than western European countries could be explained by the aforementioned differences regarding the resources invested in CKD prevention. Furthermore, exposure to hazardous living conditions and less effective public health systems to manage CKD and its complications among the less affluent eastern countries could also explain this finding [55]. The higher percentage of CKD attributed deaths and DALYs were significantly related to a higher KRT incidence. As described previously, the larger proportion of elderly in the general population among several high KRT incidence countries could explain their higher percentage of CKD attributed death and DALYs, as people live long enough to develop fatal and non-fatal CKD complications [28, 29, 56]. Notably, patients on KRT present with higher mortality rates and more impaired quality of life than patients in earlier CKD stages [57–59]. Finally, a higher prevalence of physical inactivity was also significantly related to a higher KRT incidence, a finding that remained significant after correction for multiple testing. Lower physical activity levels among high KRT incidence countries may attenuate the beneficial effects of exercise in slowing the decline in GFR [60–62], reducing adverse cardiovascular outcomes [63, 64], improving cardiorespiratory fitness [65, 66] and preventing atrophy and myopathy [67–69]. Additionally, in a large observational study in Norway, a country with low KRT incidence, it was found that, among several factors, increasing trends in physical activity were related to a stable CKD prevalence [70].

### National capacity for CKD prevention

Healthcare policies to prevent CKD and its complications by primary and secondary prevention as well as the time period in which they were implemented differ greatly among European countries [4, 6, 71]. Moreover, the impact of such policies usually requires many years to become apparent [4]. Our study showed gaps in national policies regarding the availability of guidelines for the management of main risk factors for CKD such as overweight/obesity and physical inactivity in one-third of the participating countries. We also uncovered gaps regarding the implementation of a national target on salt intake among all high KRT incidence countries. These differences in national health policies may affect the prevalence of CKD and its risk factors and therefore explain international KRT differences.

### Strengths and limitations

A main strength of this study is our extensive up-to-date data search on factors that may explain the differences in KRT incidence among almost all European countries. This article may serve as a valuable source for individual countries to compare their KRT incidence with that of other countries, and to explore which factors could potentially underlie their KRT incidence. There are also limitations to this study. First, each of the online sources for data collection had their limitations, which are described in more detail elsewhere [1, 4, 72]. For example, the Global Burden of Disease investigators have recognized important limitations regarding the complex statistical models used to address gaps when data were unavailable for many countries, including CKD attributed death and DALYs estimation processes [72]. Second, for some important factors potentially related to KRT incidence (e.g. estimated glomerular filtration rate at KRT initiation and availability of newer nephroprotective agents) data were not available (for all countries), and therefore these factors could not be included. Notably, given the timing of the regulatory approval of the sodium-glucose cotransporter-2 inhibitors for kidney protection they were not expected to influence KRT incidence values of 2021 [73]. Third, we were unable to evaluate within-country variability and we did not differentiate KRT modalities. Finally, KRT incidence data for 2021 were not available for four countries, for which we used older data.

## CONCLUSION

In this comprehensive overview of 67 factors potentially related to the KRT incidence across 38 European countries, we found that a higher number of practicing physicians, older median age at KRT initiation, a higher population density, a higher percentage of CKD attributed death and DALYs, and a higher prevalence of physical inactivity were statistically significantly related to a higher KRT incidence. After correction for multiple testing only median age at KRT initiation, population density and physical inactivity prevalence remained statistically significantly correlated with KRT incidence. Additionally, from the relationships between GDP per capita as well as CKD prevalence and KRT incidence, we observed among low KRT incidence countries, a cluster with the less affluent eastern European countries and a cluster with the wealthier western European countries, highlighting the important role of the national economic status when investigating KRT incidence differences. Our findings could serve as a source for individual countries to compare their KRT incidence and explore the factors potentially underlying their KRT incidence with that of other countries. Finally, the results of this study could be used by policy makers, stakeholders and nephrologists to optimize healthcare (planning) regarding KRT initiation and in turn reduce disparities in KRT incidence.

## Supplementary Material

gfaf163_Supplemental_Files

## Data Availability

All data supporting the findings of this study are available within the paper and its supplementary material.
