# Supplementary material for: 67 national-level factors potentially related to the incidence of kidney replacement therapy across Europe
Source: Nephrol Dial Transplant. 2025 Aug 28;41(2):309–20. doi: 10.1093/ndt/gfaf163 (PMC12855609; doi:10.1093/ndt/gfaf163)

SUPPLEMENTARY MATERIAL

Table of Contents

SUPPLEMENTARY TABLES

Table		Page
**Supplementary Table 1.**	Overview of the sources used to extract the data included in this study.	3
**Supplementary Table 3.**	The relationship between the factors significantly related to KRT incidence among wealthy (gross domestic product, GDP per capita>$40,000) and less affluent (GDP per capita<$40,000) countries.	7

SUPPLEMENTARY FIGURES

Figure		Page
**Supplementary Figure 1.**	The map with the 38 European countries included in this study.	8
**Geographical, socioeconomic and health-related factors**		
**Supplementary Figure 2.**	The relationship between geographical and socioeconomic factors and kidney replacement therapy (KRT) incidence. A. General population aged 65+ years (% of total population), B. Rural population (% of total population)	9
**Supplementary Figure 3.**	The relationship between sociocultural factors and kidney replacement therapy (KRT) incidence. A. Education Index, B. Innovation Index, C. Human Development Index	10
**Supplementary Figure 4.**	The relationship between health indicators and kidney replacement therapy (KRT) incidence. A. Life expectancy (years) at 65-69 years, B. Life expectancy (years) at 40-44 years, C. All-cause mortality rate (death rate per 100,000 population), D. Cardiovascular mortality rate (death rate per 100,000 population)	11
**Supplementary Figure 5.**	The relationship between healthcare factors and kidney replacement therapy (KRT) incidence. A. Practicing nurses (per 1,000 inhabitants), B. Nephrologists (pmp), C. Nephrology trainees (pmp), D. Salary of nephrologists per year (US$), E. Current health expenditure (% of GDP), F. Healthcare Access and Quality index, G. Total number of renal centers of country (pmp), H. Travel time to healthcare facilities	12
**Factors related to CKD**		
**Supplementary Figure 6.**	The relationship between risk factors related to chronic kidney disease (CKD) and kidney replacement therapy (KRT) incidence. A. Diabetes (%, prevalence in adults), B. Hypertension (%, prevalence in adults), C. Obesity (%, prevalence in adults), D. Smoking (%, prevalence in adults), E. Cardiovascular disease (%, prevalence in adults), F. High salt intake (g/day)	13
**Supplementary Figure 7.**	The relationship between altitude (m) and kidney replacement therapy (KRT) incidence. A. by country, b. by the most populated city of each country	14
**Supplementary Figure 8.**	Conservative care management (CCM). A. The relationship between patients who were offered CCM (%) from the EDITH study and KRT incidence, B. Capacity for CCM	15
**Supplementary Figure 9.**	The relationship between the annual cost of kidney replacement therapy (KRT) components per patient and KRT incidence. A. Hemodialysis (US$), B. Peritoneal dialysis (US$), C. Kidney Transplant (First year) (US$), D. Kidney Transplant (Later years) (US$), E. HD/PD cost ratio	16
**Factors related to national capacity for CKD prevention**		
**Supplementary Figure 10.**	Existence of evidence-based national guidelines/protocols/standards for the management of A. Overweight/obesity and B. Physical inactivity.	17
**Supplementary Figure 11.**	Existence of: A. a national target on salt and B. any policies to reduce population salt consumption.	18
**Secondary analysis according to GDP per capita**		

**Supplementary Figure 12.**	The relationship between kidney replacement therapy (KRT) incidence (pmp) and: A. Median age at KRT initiation (years), B. Physical inactivity (%, prevalence in adults), C. Practicing physicians (per 1000 inhabitants), D. Death attributed to CKD (%) and E. Disability-adjusted life years (DALYs) attributed to CKD among wealthy (GDP per capita>$40,000, yellow square marker) and less affluent (GDP per capita<$40,000, black rhombus marker) country groups.	19

Supplementary Table 1. Overview of the sources used to extract the data included in this study.

Factor	Source	Time period
KRT incidence (pmp, unadjusted)		
- Albania, Austria, Belarus, Belgium, Bosnia and Herzegovina, Croatia, Cyprus, Czech Republic, Denmark, Estonia, Finland, France, Greece, Iceland, Italy, Kosovo, Latvia, Lithuania, Montenegro, North Macedonia, Norway, Poland, Portugal, Romania, Serbia, Slovakia, Spain, Sweden, Switzerland, the Netherlands, Turkey, Ukraine and United Kingdom	ERA Registry annual report 2021 [1]	2021
- Hungary and Russia	ERA Registry annual report 2020 [2]	2020
- Bulgaria	ERA Registry annual report 2018 [3]	2018
- Georgia	ERA Registry annual report 2017 [4]	2017
- Germany (estimated KRT incidence)	Stel et al. Kidney Int. 2021 [5]	2021
**Geographical, socioeconomic and health-related factors**		
Geographical and socioeconomic factors		
Population density (people per sq.km of land area)	Worldbank.org [6]	2021
General population aged 65+ years (% of total population)	Worldbank.org [7]	2021
Rural population (% of total population)	Worldbank.org [8]	2021
GDP per capita (US$)	Worldbank.org [9]	2021
Sociocultural factors		
Education Index	Worldpopulationreview.com [10]	2021
Innovation Index	TheGlobalEconomy.com [11]	2022
Human Development Index	Human Development Reports (undp.org) [12]	2021
Health indicators		
Life expectancy (years)		
- at 65-69 years	Global Burden of Disease [13]	2021
- at 40-44 years	Global Burden of Disease [13]	2021
Mortality rate		
- all causes (death rate per 100000 population)	WHO mortality database [14]	2021
- cardiovascular (death rate per 100000 population)	WHO mortality database [15]	2021
Healthcare factors		
Practicing physicians (per 1000 inhabitants)	Worldbank.org [16]	2019-2021
Practicing nurses (per 1000 inhabitants)	Worldbank.org [17]	2019-2021
Nephrologists (pmp)	Bello et al. Lancet Glob Health. 2024 [18]	2023
Nephrology trainees (pmp)	Bello et al. Lancet Glob Health. 2024 [18]	2023
Salary of nephrologists per year (US$)	https://worldsalaries.com/ [19]	2024
Current health expenditure (% of GDP)	Worldbank.org [20]	2020-2021
Healthcare Access and Quality index	GBD 2016 Healthcare Access and Quality Collaborators. Lancet. 2018 [21]	2016
Primary care physician referral for access secondary care	OECD, Health at a Glance: Europe 2016 [22]	2016
Total number of renal centers of country (pmp) *[Calculated as: (Total number of renal centers of country)/(Total population of country)*1000000]*	Total number of renal centers of country: ERA Registry annual reports<br>- 2021 (for most countries) [1]<br>- 2020 (for Hungary and Russia) [2]	2021 (except Hungary, Russia,

	- 2018 (for Bulgaria) [3]<br>- 2017 (for Georgia) [4]<br>Total population of country: Worldbank.org [20]	Bulgaria, Georgia)
Travel time to healthcare facilities	Weiss et al. Nat Med. 2020 [23]	2019
**Factors related to CKD**		
CKD		
CKD prevalence (%)	Global Burden of Disease [13]	2021
Median age of KRT initiation (years)	ERA Registry annual report 2021 [1]	2021
Death attributed to CKD (%)	Global Burden of Disease [13]	2021
DALYS attributed to CKD (%)	Global Burden of Disease [13]	2021
Risk factors for CKD		
Diabetes (%, prevalence of diabetes in adults)	International Diabetes Federation (IDF) [24]	2021
Hypertension (%, prevalence of hypertension among adults aged 30-79 years, crude)	The Global Health Observatory (WHO) [25]	2019
Obesity (%, prevalence among adults, BMI ≥ 30, crude estimate)	The Global Health Observatory (WHO) [26]	2022
Smoking (%, adult smoking prevalence)	WHO report on the global tobacco epidemic, 2023: protect people from tobacco smoke [27]	2021
Physical inactivity (%, prevalence of among adults aged 18+ years, crude-estimate)	The Global Health Observatory (WHO) [28]	2021
Cardiovascular disease (%, prevalence in adults aged 20+ years)	Global Burden of Disease [13]	2021
High salt intake (g/day)	WHO global report on sodium intake reduction [29]	2019
Altitude (by country) (m)<br>*[We manually searched each of the 38 countries to obtain altitude information]*	Wikipedia [30]	accessed Feb. 2024
Altitude (by most populated city of each country) (m)<br>*[We manually searched the most populated city of each of the 38 countries to obtain altitude information]*	Wikipedia [30]	accessed Feb. 2024
Conservative care management (CCM)		
Capacity for CCM	2023-ISN-Global Kidney Health Atlas [31]	2019
EDITH study: CCM (among kidney failure patients)	Stel et al. Kidney Int. 2021 [5]	2021
Macroeconomic factors related to CKD		
Annual cost of KRT components per patient	2023-ISN-Global Kidney Health Atlas [31]	2022
Data about funding	2023-ISN-Global Kidney Health Atlas [31]	2022
**Factors related to national capacity for CKD prevention**		
Access to early diagnosis of CKD and risk factors (availability of procedures) at the primary health care level	The Global Health Observatory (WHO) [32]	2021
Availability of pharmacological agents in the public health sector	The Global Health Observatory (WHO) [33]	2021
Policies, strategies and action plans	The Global Health Observatory (WHO) [34]	2021

Abbreviations: BMI, body mass index; CCM, conservative care management; CKD, chronic kidney disease; DALYS, disability-adjusted life years; eGFR, estimated glomerular filtration rate; GDP, gross domestic product; HbA1c, hemoglobin A1c; KRT, kidney replacement therapy; pmp, per million population

References

1. Boerstra, B.A., et al., *The ERA Registry Annual Report 2021: a summary.* Clin Kidney J, 2024. **17**(2): p. sfad281.
2. Astley, M.E., et al., *The ERA Registry Annual Report 2020: a summary.* Clin Kidney J, 2023. **16**(8): p. 1330-1354.
3. Kramer, A., et al., *The ERA-EDTA Registry Annual Report 2018: a summary.* Clin Kidney J, 2021. **14**(1): p. 107-123.
4. Kramer, A., et al., *The ERA-EDTA Registry Annual Report 2017: a summary.* Clin Kidney J, 2020. **13**(4): p. 693-709.
5. Stel, V.S., et al., *Supplemented ERA-EDTA Registry data evaluated the frequency of dialysis, kidney transplantation, and comprehensive conservative management for patients with kidney failure in Europe.* Kidney Int, 2021. **100**(1): p. 182-195.
6. *Population density (people per sq. km of land area) | Data (worldbank.org) (https://data.worldbank.org/indicator/EN.POP.DNST?view=chart) (last assessed 30/07/2024).*
7. *Population ages 65 and above (% of total population) | Data (worldbank.org) (https://data.worldbank.org/indicator/SP.POP.65UP.TO.ZS) (last accessed 30/07/2024).*
8. *Rural population (% of total population) | Data (worldbank.org) (https://data.worldbank.org/indicator/SP.RUR.TOTL.ZS) (last accessed 30/07/2024).*
9. *GDP per capita (current US$) | Data (worldbank.org) (https://data.worldbank.org/indicator/NY.GDP.PCAP.CD) (last accessed 30/07/2024).*
10. *Education Index by Country 2024 (worldpopulationreview.com) (https://worldpopulationreview.com/country-rankings/education-index-by-country) (last accessed 30/07/2024).*
11. *Innovation index by country, around the world | TheGlobalEconomy.com (https://www.theglobaleconomy.com/rankings/gii_index/) (last accessed 30/07/2024).*
12. *Human Development Reports 23-24_Statistical_Annex_HDI_Trends_Table.xlsx (live.com) (https://view.officeapps.live.com/op/view.aspx?src=https%3A%2F%2Fhdr.undp.org%2Fsites%2Fdefault%2Ffiles%2F2023-24_HDR%2FHDR23-24_Statistical_Annex_HDI_Trends_Table.xlsx&wdOrigin=BROWSELINK) (last accessed 30/07/2024).*
13. *GBD Compare (healthdata.org) (https://vizhub.healthdata.org/gbd-compare/) (last accessed 07/06/2024).*
14. *All cause mortality (who.int) (https://platform.who.int/mortality/themes/theme-details/MDB/all-causes) (last accessed 30/07/2024).*
15. *Cardiovascular mortality (who.int) (https://platform.who.int/mortality/themes/theme-details/topics/topic-details/MDB/cardiovascular-diseases) (last accessed 30/07/2024).*
16. *Physicians (per 1,000 people) | Data (worldbank.org) (https://data.worldbank.org/indicator/SH.MED.PHYS.ZS?view=chart) (last accessed 30/07/2024).*
17. *Nurses and midwives (per 1,000 people) | Data (worldbank.org) (https://data.worldbank.org/indicator/SH.MED.NUMW.P3?view=chart) (last accessed 30/07/2024).*
18. Bello, A.K., et al., *An update on the global disparities in kidney disease burden and care across world countries and regions.* Lancet Glob Health, 2024. **12**(3): p. e382-e395.
19. *World Salaries | International Average Salary Database/Survey (https://worldsalaries.com/) (last accessed 30/07/2024).*
20. *Current health expenditure (% of GDP) | Data (worldbank.org) (https://data.worldbank.org/indicator/SH.XPD.CHEX.GD.ZS?view=chart) (last accessed 30/07/2024).*
21. *Measuring performance on the Healthcare Access and Quality Index for 195 countries and territories and selected subnational locations: a systematic analysis from the Global Burden of Disease Study 2016.* Lancet, 2018. **391**(10136): p. 2236-2271.
22. OECD and E. Union, *Health at a Glance: Europe 2016 (https://doi.org/10.1787/9789264265592-en) (last accessed 30/07/2024).* 2016.
23. Weiss, D.J., et al., *Global maps of travel time to healthcare facilities.* Nat Med, 2020. **26**(12): p. 1835-1838.
24. *International Diabetes Federation | Europe Site (https://idf.org/europe/our-network/our-members/) (last accessed 30/07/2024).*

25. *Prevalence of hypertension among adults aged 30-79 years (who.int) (https://www.who.int/data/gho/data/indicators/indicator-details/GHO/prevalence-of-hypertension-among-adults-aged-30-79-years) (last accessed 30/07/2024).*
26. *Prevalence of obesity among adults, BMI >= 30 (crude estimate) (%) (who.int) (https://www.who.int/data/gho/data/indicators/indicator-details/GHO/prevalence-of-obesity-among-adults-bmi-=-30-(crude-estimate)-(-)) (last accessed 30/07/2024).*
27. *WHO report on the global tobacco epidemic, 2023: protect people from tobacco smoke (https://www.who.int/publications/i/item/9789240077164) (last accessed 30/07/2024).*
28. *Prevalence of insufficient physical activity among adults aged 18+ years (crude estimate) (%) (who.int) (https://www.who.int/data/gho/data/indicators/indicator-details/GHO/prevalence-of-insufficient-physical-activity-among-adults-aged-18-years-(crude-estimate)-(-)) (last accessed 30/07/2024).*
29. *WHO global report on sodium intake reduction (https://www.who.int/publications/i/item/9789240069985) (last accessed 30/07/2024).*
30. *Wikipedia, the free encyclopedia (https://en.wikipedia.org/wiki/Main_Page) (last accessed 30/07/2024).*
31. *Bello, A.K., et al., (2023). ISN–Global Kidney Health Atlas: A report by the International Society of Nephrology: An Assessment of Global Kidney Health Care Status focusing on Capacity, Availability, Accessibility, Affordability and Outcomes of Kidney Disease. International Society of Nephrology, Brussels, Belgium.*
32. *NCD-related tests and procedures in primary health care (who.int) (https://www.who.int/data/gho/data/themes/topics/indicator-groups/indicator-group-details/GHO/related-tests-and-procedures-in-primary-health-care) (last accessed 30/07/2024).*
33. *NCD-related medicines (who.int) (https://www.who.int/data/gho/data/themes/topics/indicator-groups/indicator-group-details/GHO/related-medicines) (last accessed 30/07/2024).*
34. *NCD national capacity: Management of NCDs: Guidelines (who.int) (https://www.who.int/data/gho/data/themes/topics/indicator-groups/indicator-group-details/GHO/management-of-ncds---guidelines) (last accessed 30/07/2024).*

Supplementary Table 3. The relationship between the factors significantly related to KRT incidence among wealthy (GDP per capita>$40,000) and less affluent (GDP per capita<$40,000) countries.

Factor	Less affluent countries (GDP per capita < $40,000) (26 countries)		Wealthy countries (GDP per capita > $40,000) (12 countries)	
	Mean (standard deviation) or Median (interquartile range)	Relationship (for less affluent countries)	Mean (standard deviation) or Median (interquartile range)	Relationship (for wealthy countries)
Median age at KRT initiation (years)	65.5 (4.3)	R=0.684, **p<0.001**	68.4 (2.2)	R=0.889, **p<0.001***
Physical inactivity (%, prevalence in adults)	27.0 (23.9-39.7)	R=0.608, **p=0.006***	21.0 (13.1-26.6)	R=0.316, p=0.623*
Practicing physicians (per 1,000 inhabitants)	3.9 (1.2)	R=0.734, **p<0.001***	4.7 (1.2)	R=0.255, p=0.739*
Population density (people per sq.km of land area)	82 (59-113)	R=0.535, **p=0.005**	135 (20-267)	R=0.473, p=0.121
Death attributed to CKD (%)	1.4 (1.0-2.8)	R=0.550, **p=0.019***	2.6 (1.8-3.6)	R=0.456, p=0.351*
DALYs attributed to CKD (%)	1.3 (1.0-1.7)	R=0.495, **p=0.012**	1.6 (1.2-1.9)	R=0.470, p=0.325*

Values are expressed as mean (standard deviation) or median (interquartile range) according to their distribution (continuous factors).
The R coefficient represents the Pearson's (for normally distributed data) or Spearman's (for non-normally distributed data) correlation coefficient for simple linear relationships and the multiple correlation coefficient for curvilinear relationships including all individual countries with GDP per capita < $40,000 and > $40,000 respectively. Curvilinear relationships are indicated with *.

Abbreviations: CKD, chronic kidney disease; DALYs, disability-adjusted life years; GDP, gross domestic product; KRT, kidney replacement therapy.

Supplementary Figure 1. The map with the 38 European countries included in this study, stratified in groups of low- (0-100 pmp, green color), middle- (100-200 pmp, yellow color) and high- (>200 pmp, blue color) kidney replacement therapy (KRT) incidence groups.

KRT incidence, unadjusted (pmp)

Low (0-100)	
UA	Ukraine
RS	Serbia
BY	Belarus
EE	Estonia
ME	Montenegro
FI	Finland
RU	Russia
IS	Iceland
CH	Switzerland
LV	Latvia
LT	Lithuania
NO	Norway

Middle (100-200)	
SE	Sweden
GB	United Kingdom
NL	The Netherlands
AT	Austria
BA	Bosnia and Herzegovina
DK	Denmark
HR	Croatia
AL	Albania
PL	Poland
TR	Turkey
ES	Spain
BG	Bulgaria
XK	Kosovo
DE	Germany
IT	Italy
FR	France
RO	Romania
MK	North Macedonia
SK	Slovakia
BE	Belgium
HU	Hungary

High (>200)	
GE	Georgia
CZ	Czech Republic
PT	Portugal
GR	Greece
CY	Cyprus

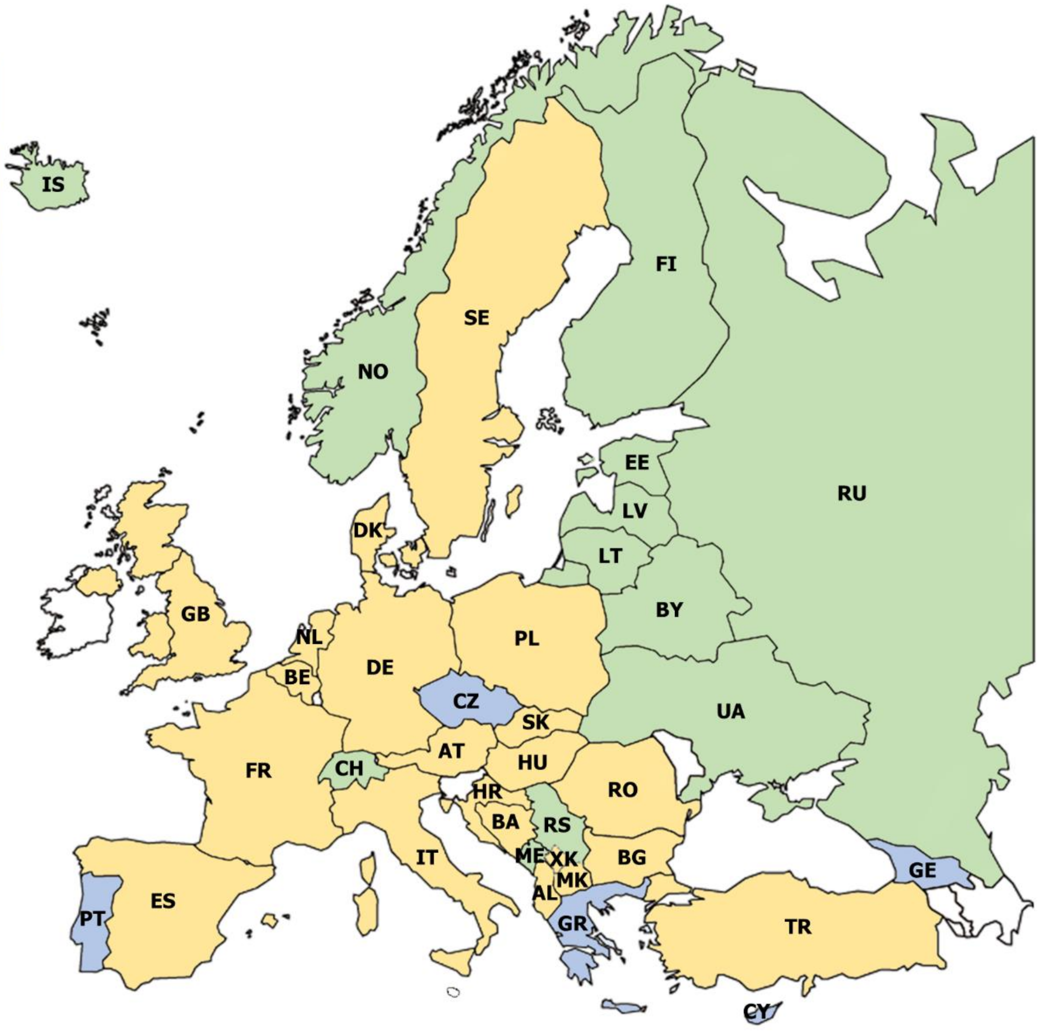

Supplementary Figure 2. The relationship between geographical and socioeconomic factors and kidney replacement therapy (KRT) incidence. A. General population aged 65+ years (% of total population), B. Rural population (% of total population)

Countries are stratified: a. by KRT incidence, in low- (0-100 pmp, green color), middle- (100-200 pmp, yellow color) and high- (>200 pmp, blue color) incidence groups, b. by GDP per capita, in wealthy (GDP per capita>$40,000, yellow square marker) and less affluent (GDP per capita<$40,000, black rhombus marker) country groups.

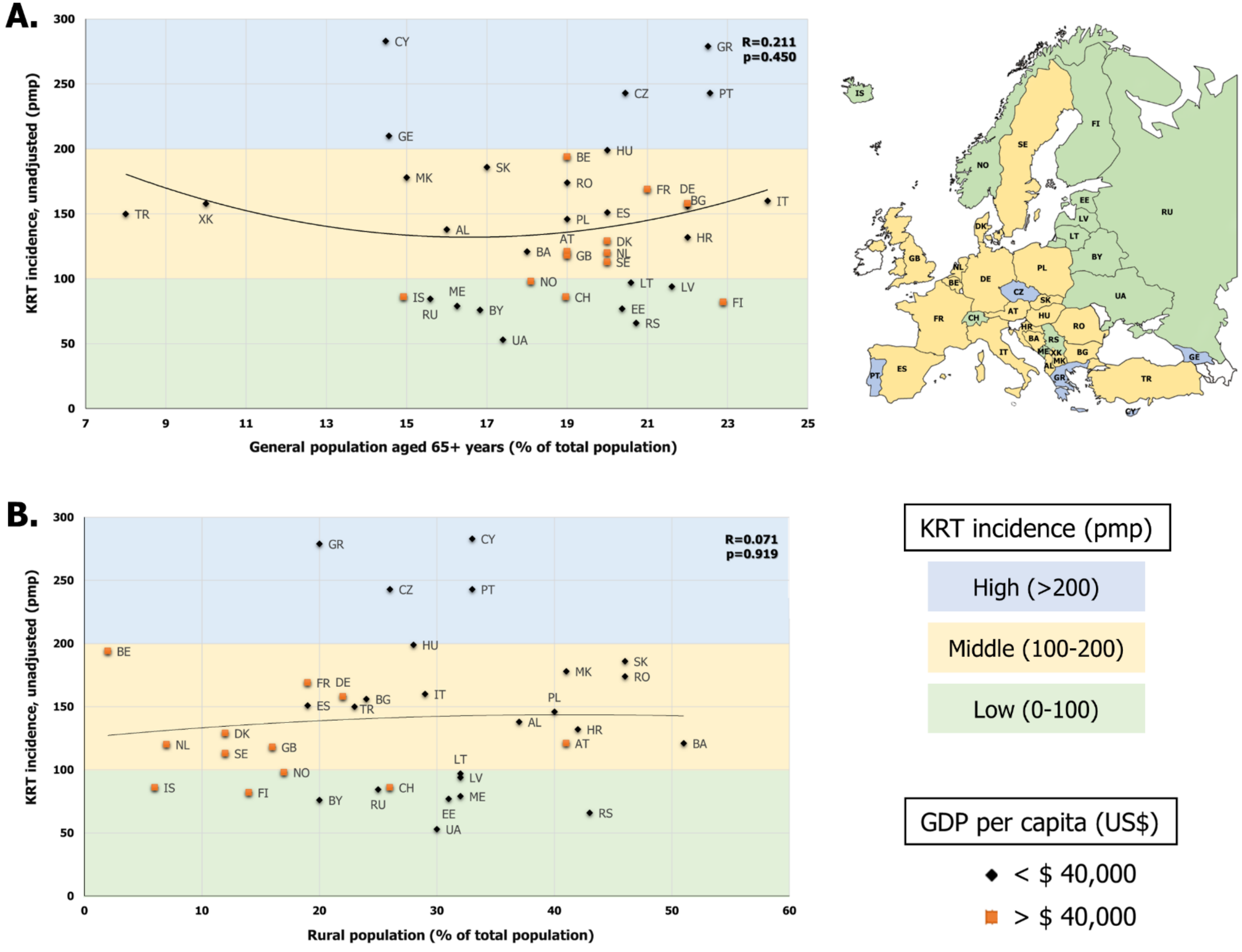

Supplementary Figure 3. The relationship between sociocultural factors and kidney replacement therapy (KRT) incidence. A. Education Index, B. Innovation Index, C. Human Development Index

Countries are stratified: a. by KRT incidence, in low- (0-100 pmp, green color), middle- (100-200 pmp, yellow color) and high- (>200 pmp, blue color) incidence groups, b. by GDP per capita, in wealthy (GDP per capita>$40,000, yellow square marker) and less affluent (GDP per capita<$40,000, black rhombus marker) country groups.

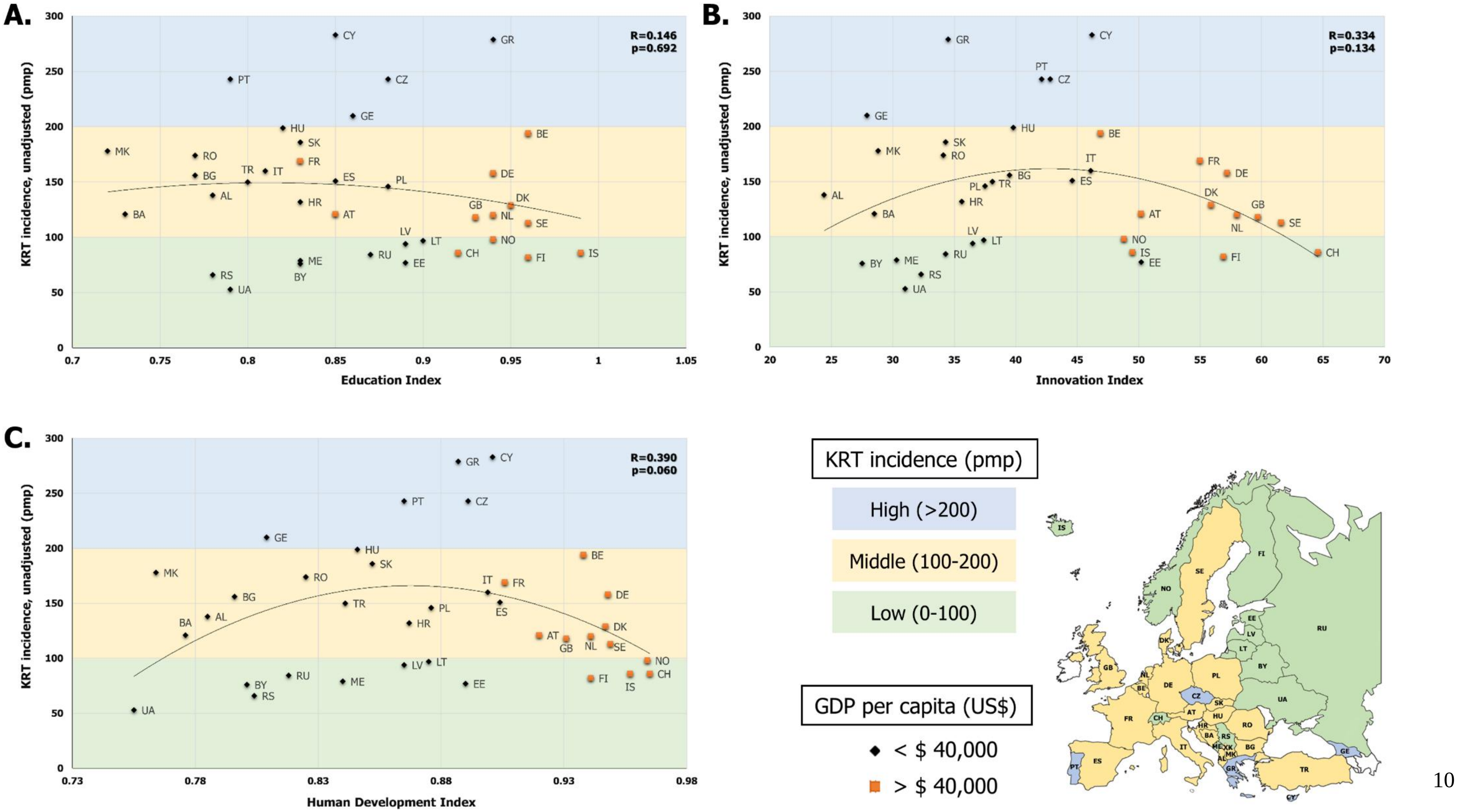

Supplementary Figure 4. The relationship between health indicators and kidney replacement therapy (KRT) incidence. A. Life expectancy (years) at 65-69 years, B. Life expectancy (years) at 40-44 years, C. All-cause mortality rate (death rate per 100,000 population), D. Cardiovascular mortality rate (death rate per 100,000 population)

Countries are stratified: a. by KRT incidence, in low- (0-100 pmp, green color), middle- (100-200 pmp, yellow color) and high- (>200 pmp, blue color) incidence groups, b. by GDP per capita, in wealthy (GDP per capita>$40,000, yellow square marker) and less affluent (GDP per capita<$40,000, black rhombus marker) country groups.

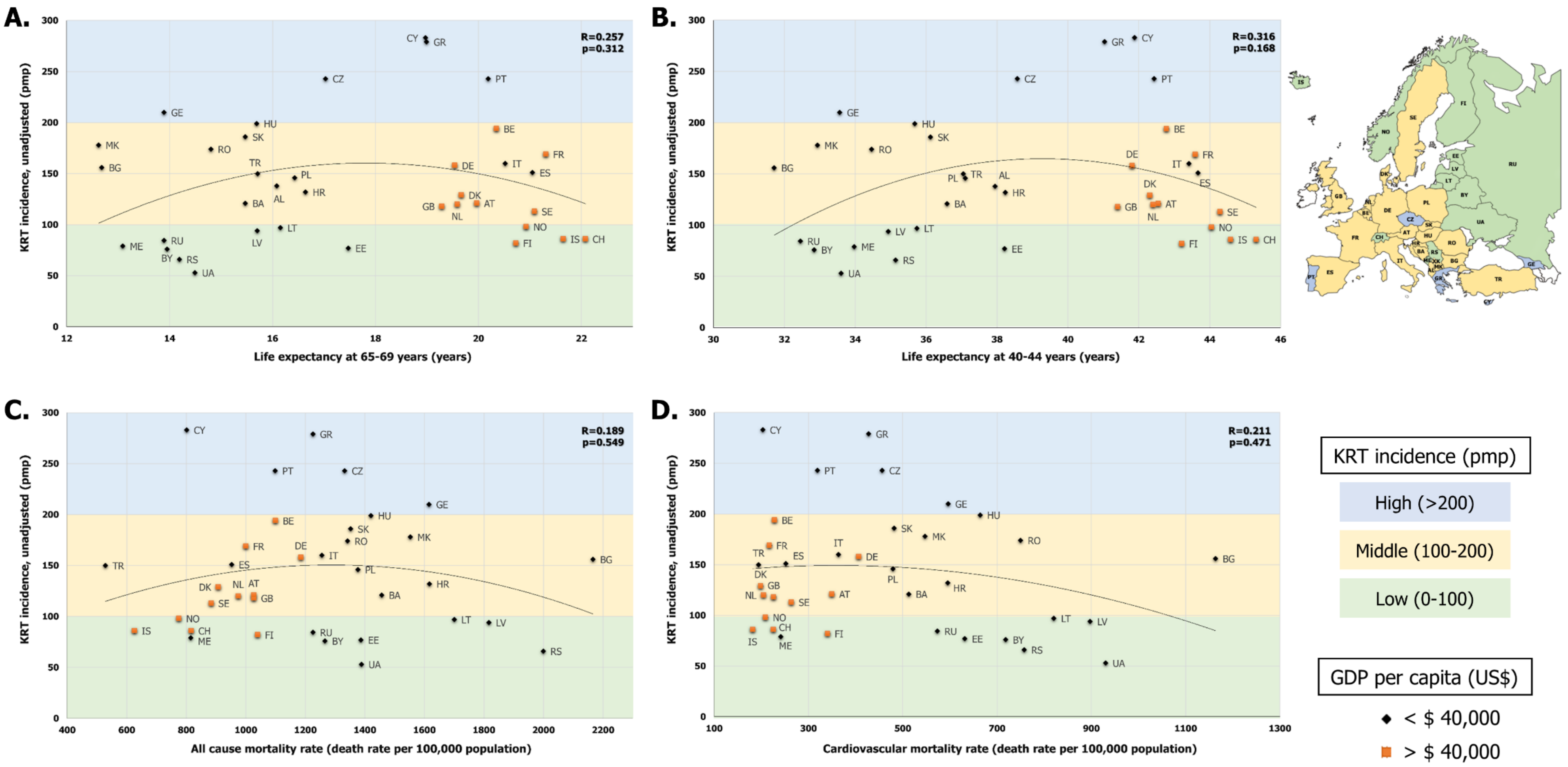

Supplementary Figure 5. The relationship between healthcare factors and kidney replacement therapy (KRT) incidence. A. Practicing nurses (per 1,000 inhabitants), B. Nephrologists (pmp), C. Nephrology trainees (pmp), D. Salary of nephrologists per year (US$), E. Current health expenditure (% of GDP), F. Healthcare Access and Quality index, G. Total number of renal centers of country (pmp), H. Travel time to healthcare facilities

Countries are stratified: a. by KRT incidence, in low- (0-100 pmp, green color), middle- (100-200 pmp, yellow color) and high- (>200 pmp, blue color) incidence groups, b. by GDP per capita, in wealthy (GDP per capita>$40,000, yellow square marker) and less affluent (GDP per capita<$40,000, black rhombus marker) country groups.

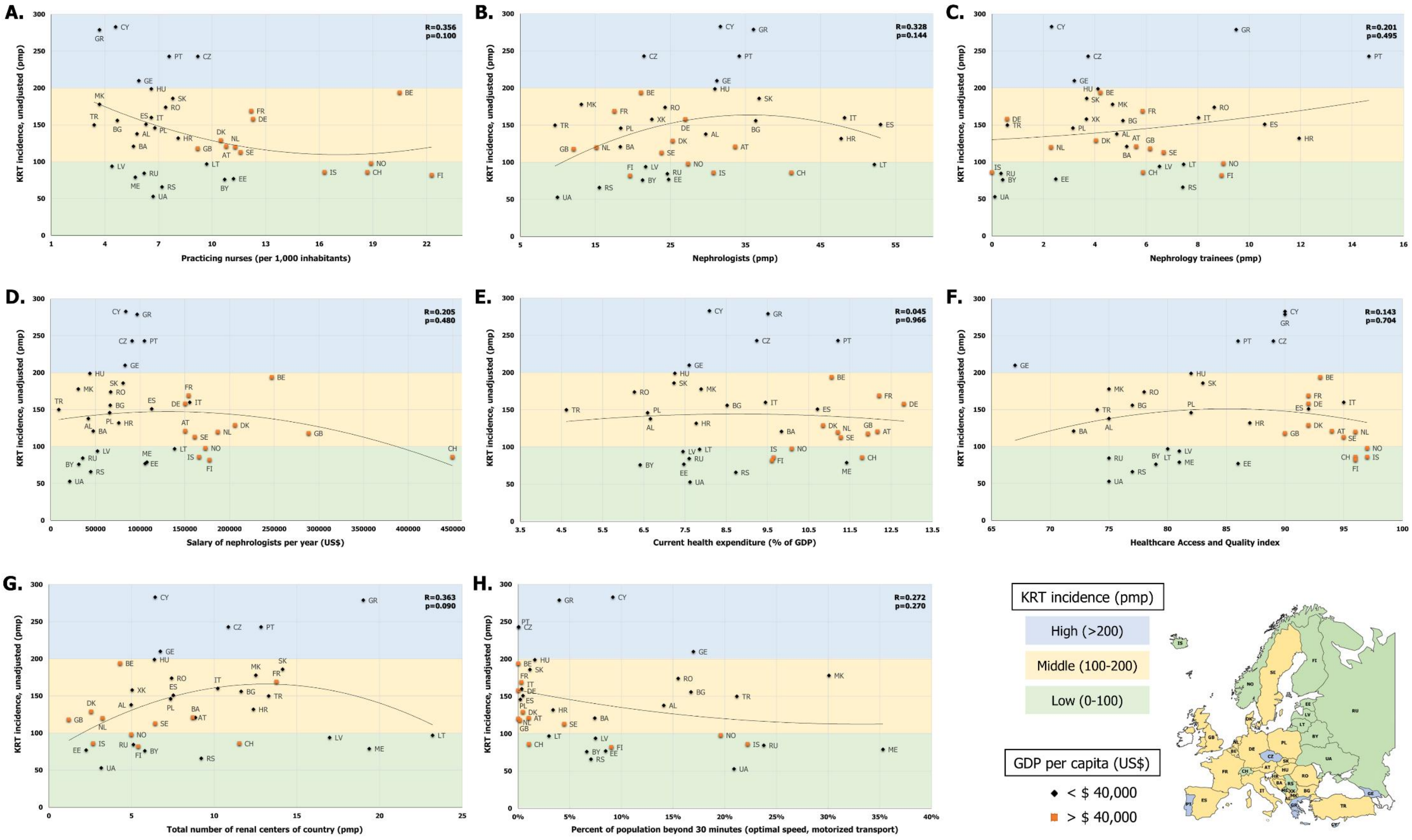

Supplementary Figure 6. The relationship between risk factors related to chronic kidney disease (CKD) and kidney replacement therapy (KRT) incidence. A. Diabetes (%, prevalence in adults), B. Hypertension (%, prevalence in adults), C. Obesity (%, prevalence in adults), D. Smoking (%, prevalence in adults), E. Cardiovascular disease (%, prevalence in adults), F. High salt intake (g/day)

Countries are stratified: a. by KRT incidence, in low- (0-100 pmp, green color), middle- (100-200 pmp, yellow color) and high- (>200 pmp, blue color) incidence groups, b. by GDP per capita, in wealthy (GDP per capita>$40,000, yellow square marker) and less affluent (GDP per capita<$40,000, black rhombus marker) country groups.

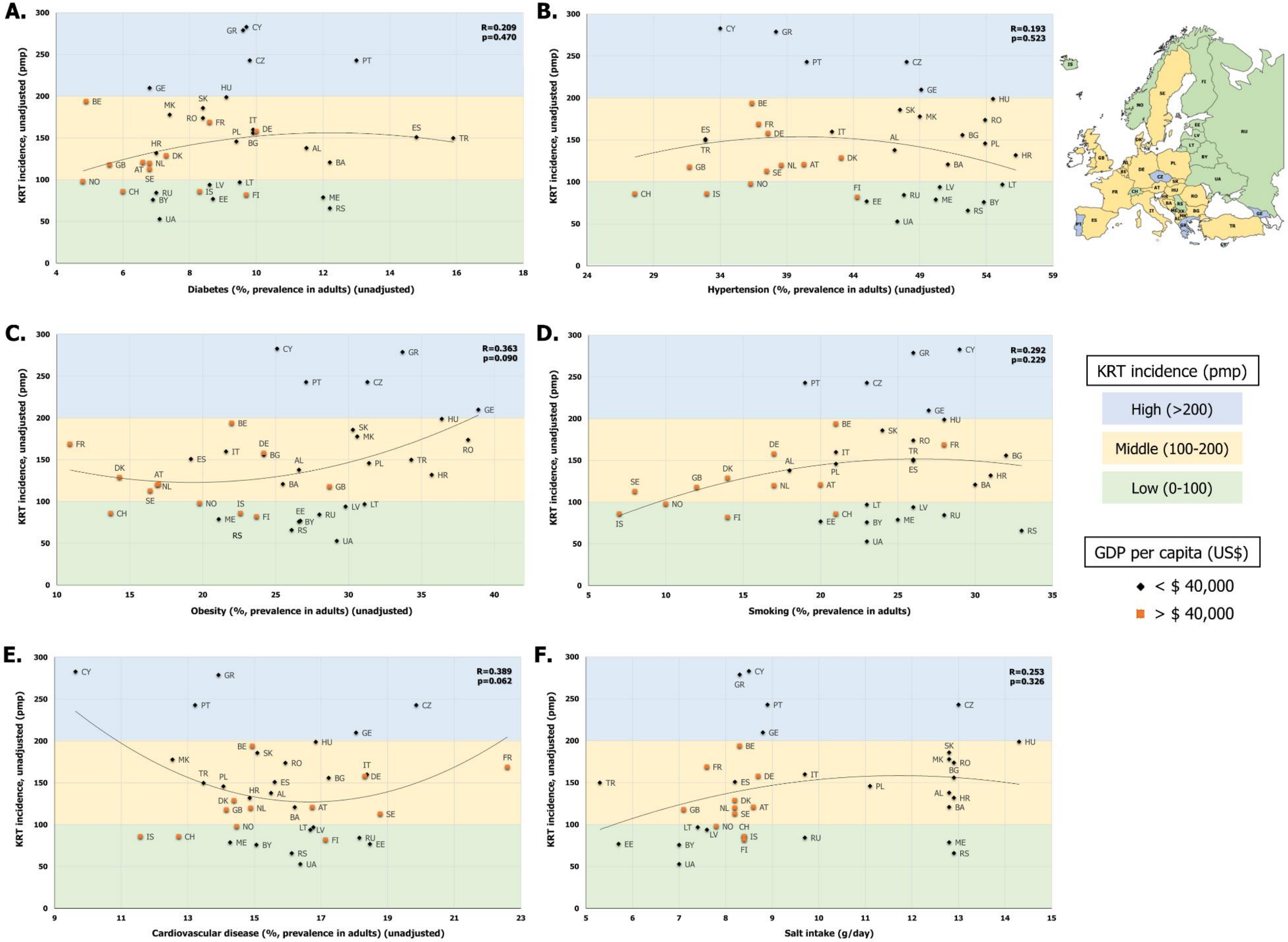

Supplementary Figure 7. The relationship between altitude (m) and kidney replacement therapy (KRT) incidence. A. by country, B. by the most populated city of each country

Countries are stratified: a. by KRT incidence, in low- (0-100 pmp, green color), middle- (100-200 pmp, yellow color) and high- (>200 pmp, blue color) incidence groups, b. by GDP per capita, in wealthy (GDP per capita>$40,000, yellow square marker) and less affluent (GDP per capita<$40,000, black rhombus marker) country groups.

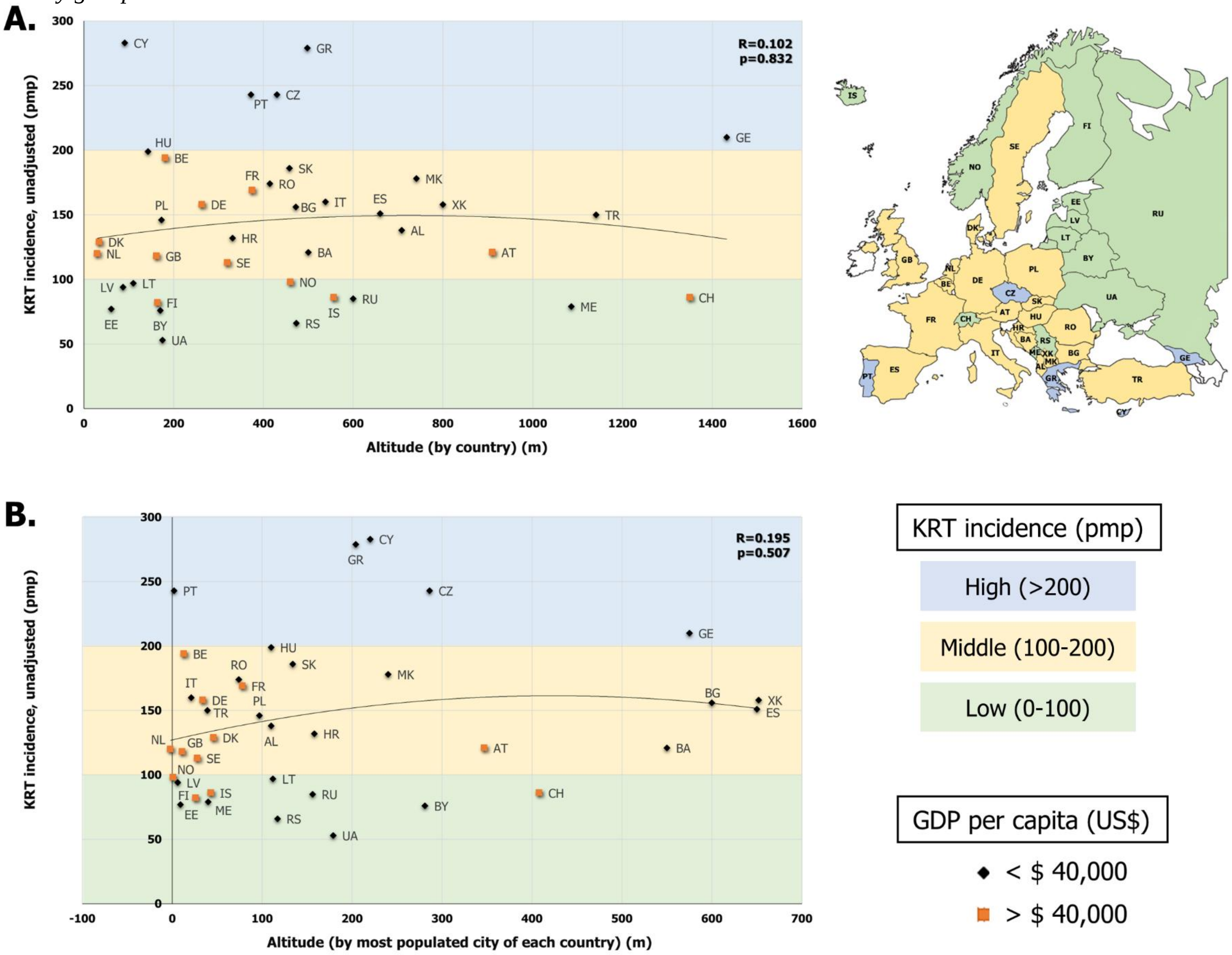

Supplementary Figure 8. Conservative care management (CCM). A. The relationship between patients who were offered CCM (%) from the EDITH study and KRT incidence, B. Capacity for CCM

Countries are stratified: a. by KRT incidence, in low- (0-100 pmp, green color), middle- (100-200 pmp, yellow color) and high- (>200 pmp, blue color) incidence groups, b. by GDP per capita, in wealthy (GDP per capita>$40,000) and less affluent (GDP per capita<$40,000) country groups.

A. Median % of patients who were offered conservative care management

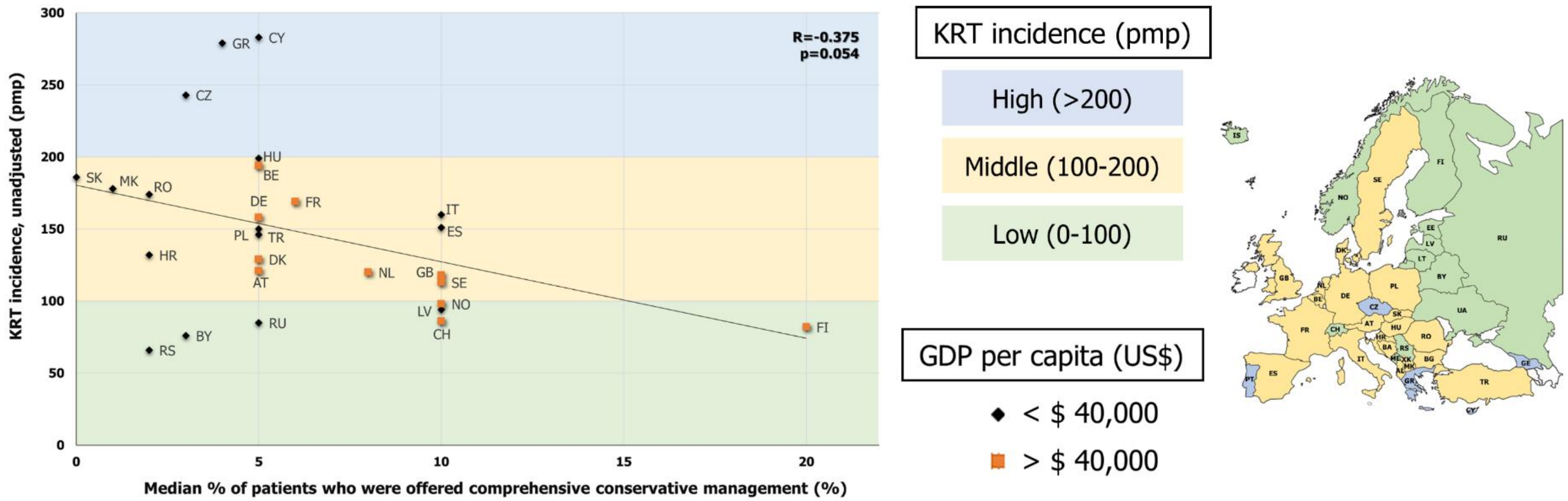

B. Capacity for conservative care management

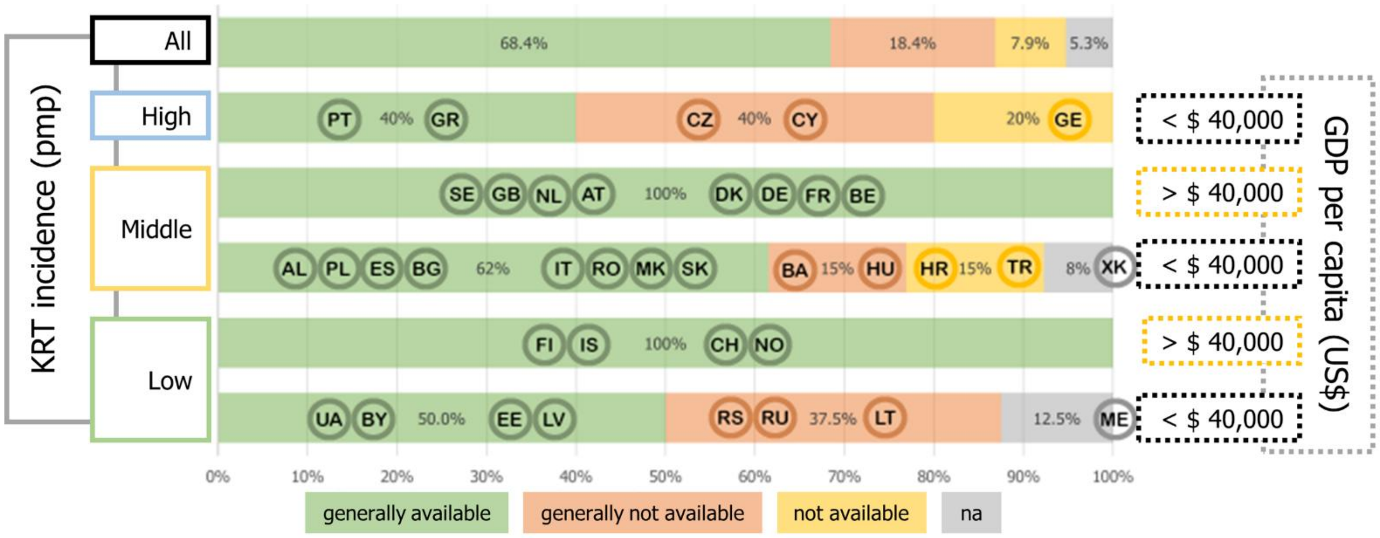

Supplementary Figure 9. The relationship between the annual cost of kidney replacement therapy (KRT) components per patient and KRT incidence. A. Hemodialysis (US$), B. Peritoneal dialysis (US$), C. Kidney Transplant (First year) (US$), D. Kidney Transplant (Later years) (US$), E. HD/PD cost ratio

Countries are stratified: a. by KRT incidence, in low- (0-100 pmp, green color), middle- (100-200 pmp, yellow color) and high- (>200 pmp, blue color) incidence groups, b. by GDP per capita, in wealthy (GDP per capita>$40,000, yellow square marker) and less affluent (GDP per capita<$40,000, black rhombus marker) country groups.

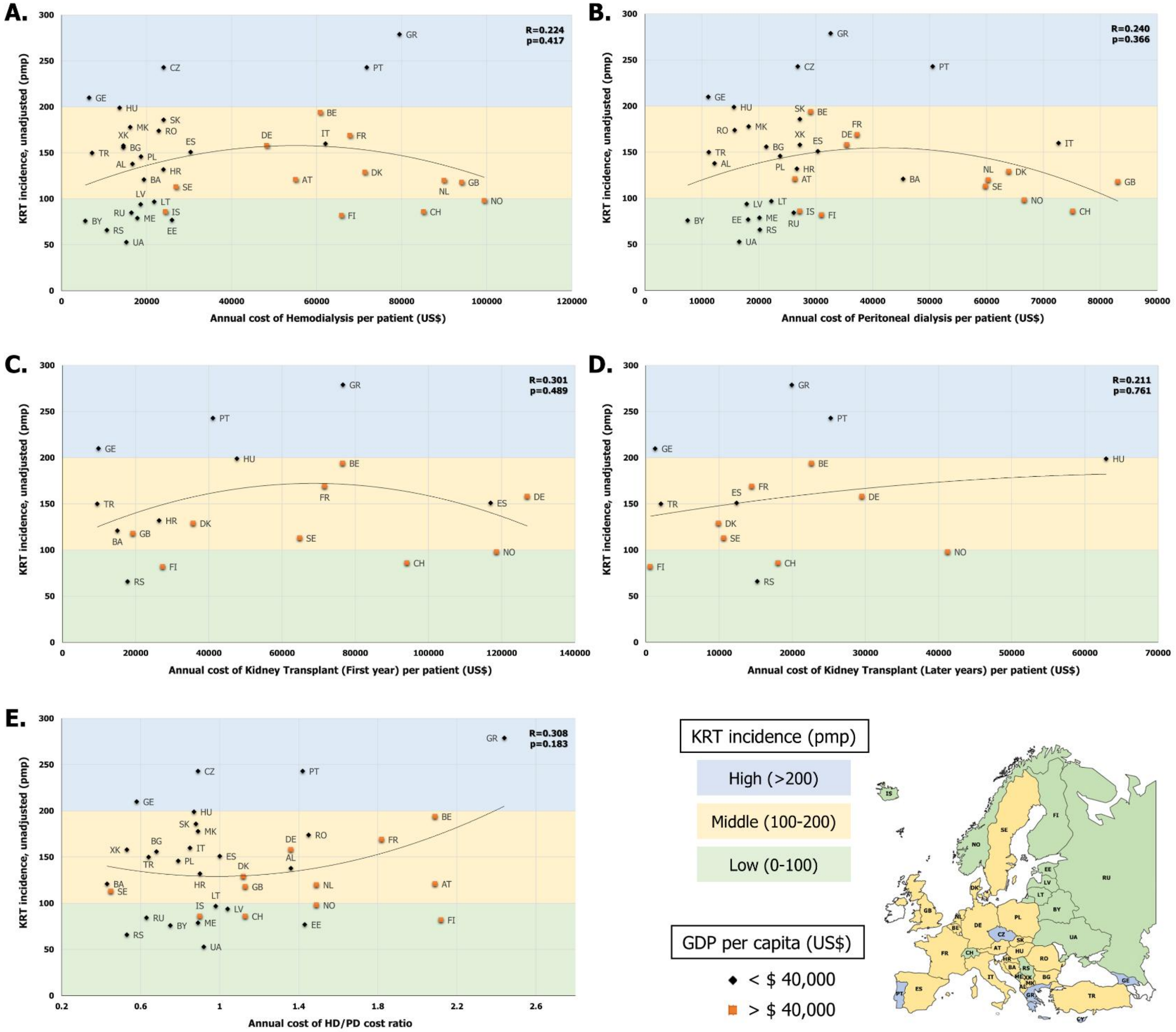

Supplementary Figure 10. Existence of evidence-based national guidelines/protocols/standards for the management of A. Overweight/obesity and B. Physical inactivity.

Countries are stratified: a. by KRT incidence, in low- (0-100 pmp), middle- (100-200 pmp) and high- (>200 pmp) incidence groups, b. by GDP per capita, in wealthy (GDP per capita>$40,000) and less affluent (GDP per capita<$40,000) country groups.

A. Guidelines for overweight/obesity

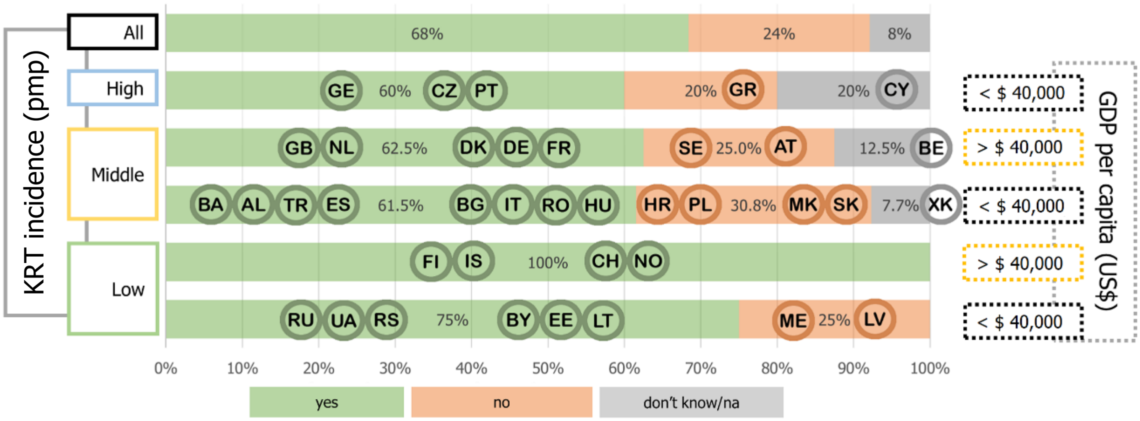

B. Guidelines for physical inactivity

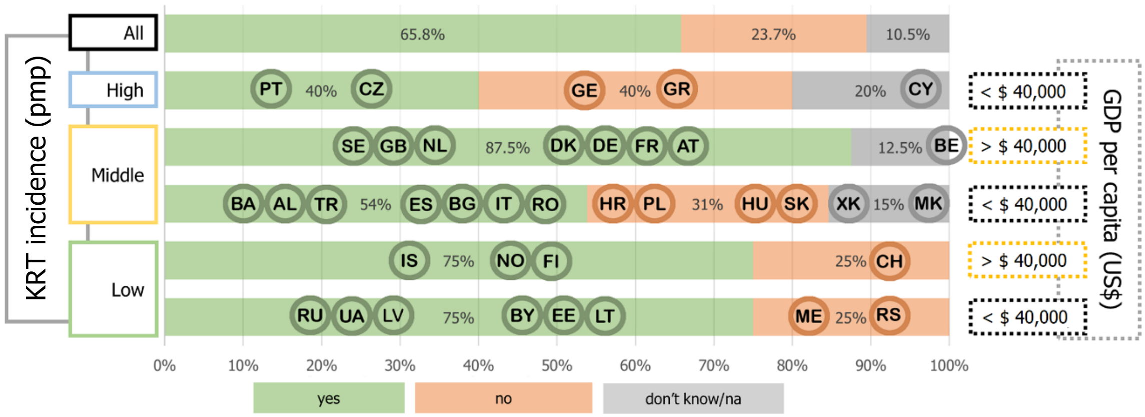

Supplementary Figure 11. Existence of: A. a national target on salt and B. any policies to reduce population salt consumption.

Countries are stratified: a. by KRT incidence, in low- (0-100 pmp), middle- (100-200 pmp) and high- (>200 pmp) incidence groups, b. by GDP per capita, in wealthy (GDP per capita>$40,000) and less affluent (GDP per capita<$40,000) country groups.

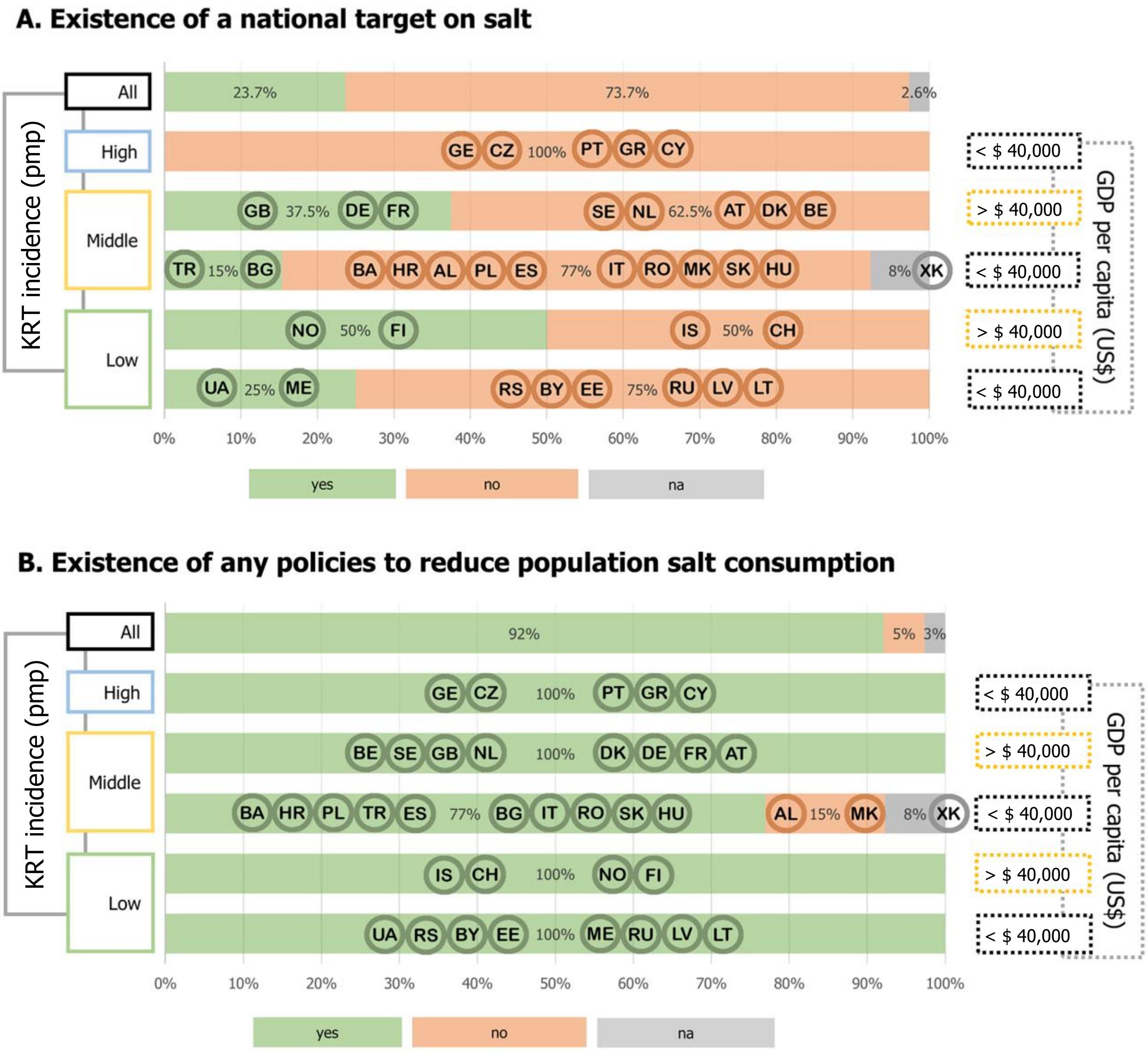

Supplementary Figure 12. The relationship between kidney replacement therapy (KRT) incidence (pmp) and: A. Median age at KRT initiation (years), B. Physical inactivity (%, prevalence in adults), C. Practicing physicians (per 1,000 inhabitants), D. Death attributed to CKD (%) and E. Disability-adjusted life years (DALYs) attributed to CKD among wealthy (GDP per capita>$40,000) and less affluent (GDP per capita<$40,000) country groups.

Countries are stratified: a. by KRT incidence, in low- (0-100 pmp, green color), middle- (100-200 pmp, yellow color) and high- (>200 pmp, blue color) incidence groups, b. by GDP per capita, in wealthy (GDP per capita>$40,000, yellow square marker) and less affluent (GDP per capita<$40,000, black rhombus marker) country groups.

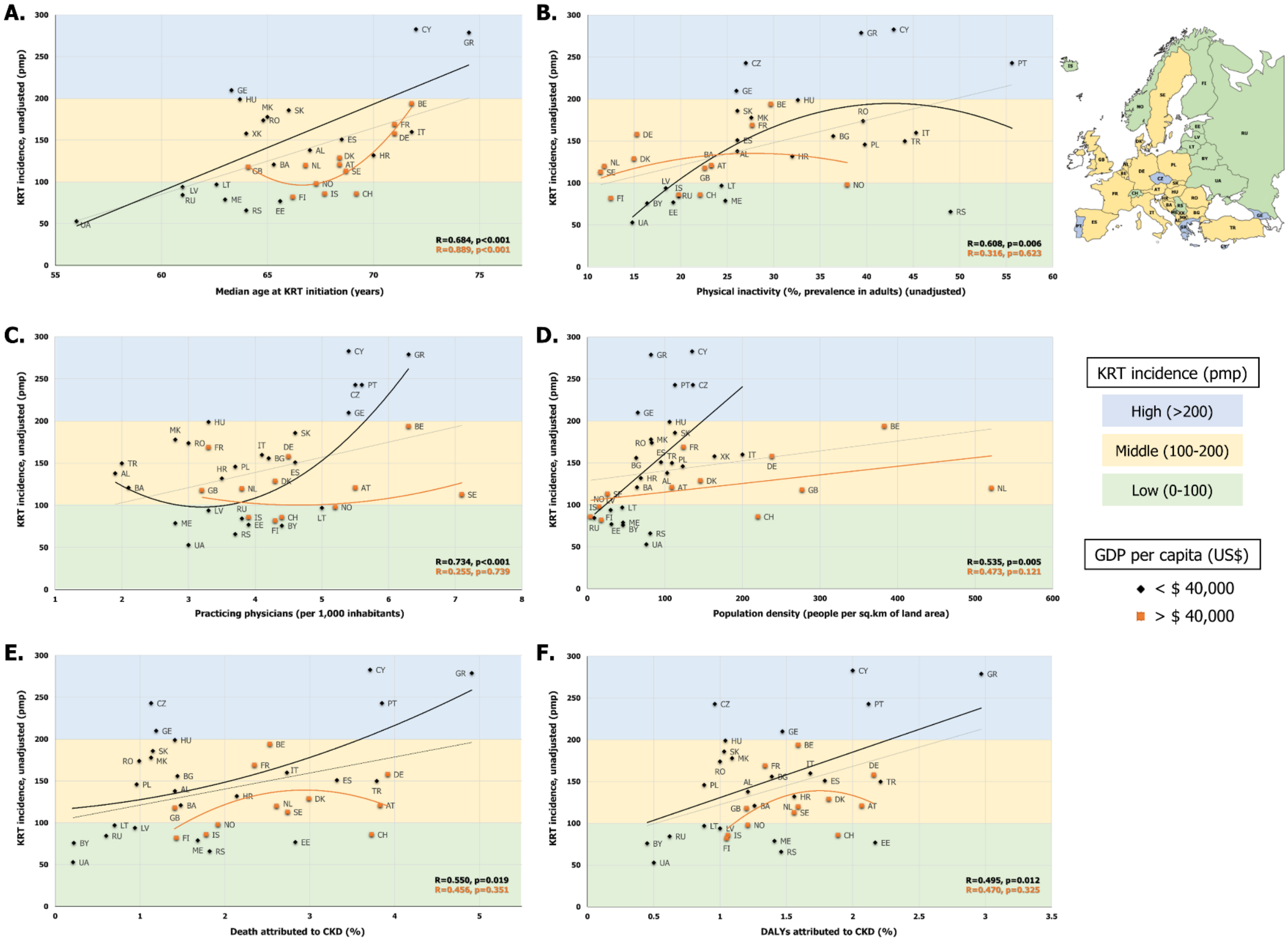

Supplement: gfaf163_Supplemental_Files [file gfaf163_supplemental_files.zip › R1.Supplementary Material.pdf]
